# Measurement of detector-corrected observables sensitive to the anomalous production of events with jets and large missing transverse momentum in $\varvec{pp}$ collisions at $\mathbf {\sqrt{s}=13}$ TeV using the ATLAS detector

**DOI:** 10.1140/epjc/s10052-017-5315-6

**Published:** 2017-11-15

**Authors:** M. Aaboud, G. Aad, B. Abbott, J. Abdallah, O. Abdinov, B. Abeloos, S. H. Abidi, O. S. AbouZeid, N. L. Abraham, H. Abramowicz, H. Abreu, R. Abreu, Y. Abulaiti, B. S. Acharya, S. Adachi, L. Adamczyk, J. Adelman, M. Adersberger, T. Adye, A. A. Affolder, T. Agatonovic-Jovin, C. Agheorghiesei, J. A. Aguilar-Saavedra, S. P. Ahlen, F. Ahmadov, G. Aielli, S. Akatsuka, H. Akerstedt, T. P. A. Åkesson, E. Akilli, A. V. Akimov, G. L. Alberghi, J. Albert, P. Albicocco, M. J. Alconada Verzini, M. Aleksa, I. N. Aleksandrov, C. Alexa, G. Alexander, T. Alexopoulos, M. Alhroob, B. Ali, M. Aliev, G. Alimonti, J. Alison, S. P. Alkire, B. M. M. Allbrooke, B. W. Allen, P. P. Allport, A. Aloisio, A. Alonso, F. Alonso, C. Alpigiani, A. A. Alshehri, M. I. Alstaty, B. Alvarez Gonzalez, D. Álvarez Piqueras, M. G. Alviggi, B. T. Amadio, Y. Amaral Coutinho, C. Amelung, D. Amidei, S. P. Amor Dos Santos, A. Amorim, S. Amoroso, G. Amundsen, C. Anastopoulos, L. S. Ancu, N. Andari, T. Andeen, C. F. Anders, J. K. Anders, K. J. Anderson, A. Andreazza, V. Andrei, S. Angelidakis, I. Angelozzi, A. Angerami, A. V. Anisenkov, N. Anjos, A. Annovi, C. Antel, M. Antonelli, A. Antonov, D. J. Antrim, F. Anulli, M. Aoki, L. Aperio Bella, G. Arabidze, Y. Arai, J. P. Araque, V. Araujo Ferraz, A. T. H. Arce, R. E. Ardell, F. A. Arduh, J.-F. Arguin, S. Argyropoulos, M. Arik, A. J. Armbruster, L. J. Armitage, O. Arnaez, H. Arnold, M. Arratia, O. Arslan, A. Artamonov, G. Artoni, S. Artz, S. Asai, N. Asbah, A. Ashkenazi, L. Asquith, K. Assamagan, R. Astalos, M. Atkinson, N. B. Atlay, K. Augsten, G. Avolio, B. Axen, M. K. Ayoub, G. Azuelos, A. E. Baas, M. J. Baca, H. Bachacou, K. Bachas, M. Backes, M. Backhaus, P. Bagnaia, H. Bahrasemani, J. T. Baines, M. Bajic, O. K. Baker, E. M. Baldin, P. Balek, F. Balli, W. K. Balunas, E. Banas, Sw. Banerjee, A. A. E. Bannoura, L. Barak, E. L. Barberio, D. Barberis, M. Barbero, T. Barillari, M.-S. Barisits, J. T. Barkeloo, T. Barklow, N. Barlow, S. L. Barnes, B. M. Barnett, R. M. Barnett, Z. Barnovska-Blenessy, A. Baroncelli, G. Barone, A. J. Barr, L. Barranco Navarro, F. Barreiro, J. Barreiro Guimarães da Costa, R. Bartoldus, A. E. Barton, P. Bartos, A. Basalaev, A. Bassalat, R. L. Bates, S. J. Batista, J. R. Batley, M. Battaglia, M. Bauce, F. Bauer, H. S. Bawa, J. B. Beacham, M. D. Beattie, T. Beau, P. H. Beauchemin, P. Bechtle, H. P. Beck, K. Becker, M. Becker, M. Beckingham, C. Becot, A. J. Beddall, A. Beddall, V. A. Bednyakov, M. Bedognetti, C. P. Bee, T. A. Beermann, M. Begalli, M. Begel, J. K. Behr, A. S. Bell, G. Bella, L. Bellagamba, A. Bellerive, M. Bellomo, K. Belotskiy, O. Beltramello, N. L. Belyaev, O. Benary, D. Benchekroun, M. Bender, K. Bendtz, N. Benekos, Y. Benhammou, E. Benhar Noccioli, J. Benitez, D. P. Benjamin, M. Benoit, J. R. Bensinger, S. Bentvelsen, L. Beresford, M. Beretta, D. Berge, E. Bergeaas Kuutmann, N. Berger, J. Beringer, S. Berlendis, N. R. Bernard, G. Bernardi, C. Bernius, F. U. Bernlochner, T. Berry, P. Berta, C. Bertella, G. Bertoli, F. Bertolucci, I. A. Bertram, C. Bertsche, D. Bertsche, G. J. Besjes, O. Bessidskaia Bylund, M. Bessner, N. Besson, C. Betancourt, A. Bethani, S. Bethke, A. J. Bevan, J. Beyer, R. M. Bianchi, O. Biebel, D. Biedermann, R. Bielski, N. V. Biesuz, M. Biglietti, J. Bilbao De Mendizabal, T. R. V. Billoud, H. Bilokon, M. Bindi, A. Bingul, C. Bini, S. Biondi, T. Bisanz, C. Bittrich, D. M. Bjergaard, C. W. Black, J. E. Black, K. M. Black, R. E. Blair, T. Blazek, I. Bloch, C. Blocker, A. Blue, W. Blum, U. Blumenschein, S. Blunier, G. J. Bobbink, V. S. Bobrovnikov, S. S. Bocchetta, A. Bocci, C. Bock, M. Boehler, D. Boerner, D. Bogavac, A. G. Bogdanchikov, C. Bohm, V. Boisvert, P. Bokan, T. Bold, A. S. Boldyrev, A. E. Bolz, M. Bomben, M. Bona, M. Boonekamp, A. Borisov, G. Borissov, J. Bortfeldt, D. Bortoletto, V. Bortolotto, D. Boscherini, M. Bosman, J. D. Bossio Sola, J. Boudreau, J. Bouffard, E. V. Bouhova-Thacker, D. Boumediene, C. Bourdarios, S. K. Boutle, A. Boveia, J. Boyd, I. R. Boyko, J. Bracinik, A. Brandt, G. Brandt, O. Brandt, U. Bratzler, B. Brau, J. E. Brau, W. D. Breaden Madden, K. Brendlinger, A. J. Brennan, L. Brenner, R. Brenner, S. Bressler, D. L. Briglin, T. M. Bristow, D. Britton, D. Britzger, F. M. Brochu, I. Brock, R. Brock, G. Brooijmans, T. Brooks, W. K. Brooks, J. Brosamer, E. Brost, J. H Broughton, P. A. Bruckman de Renstrom, D. Bruncko, A. Bruni, G. Bruni, L. S. Bruni, B. H. Brunt, M. Bruschi, N. Bruscino, P. Bryant, L. Bryngemark, T. Buanes, Q. Buat, P. Buchholz, A. G. Buckley, I. A. Budagov, F. Buehrer, M. K. Bugge, O. Bulekov, D. Bullock, T. J. Burch, H. Burckhart, S. Burdin, C. D. Burgard, A. M. Burger, B. Burghgrave, K. Burka, S. Burke, I. Burmeister, J. T. P. Burr, E. Busato, D. Büscher, V. Büscher, P. Bussey, J. M. Butler, C. M. Buttar, J. M. Butterworth, P. Butti, W. Buttinger, A. Buzatu, A. R. Buzykaev, S. Cabrera Urbán, D. Caforio, V. M. Cairo, O. Cakir, N. Calace, P. Calafiura, A. Calandri, G. Calderini, P. Calfayan, G. Callea, L. P. Caloba, S. Calvente Lopez, D. Calvet, S. Calvet, T. P. Calvet, R. Camacho Toro, S. Camarda, P. Camarri, D. Cameron, R. Caminal Armadans, C. Camincher, S. Campana, M. Campanelli, A. Camplani, A. Campoverde, V. Canale, M. Cano Bret, J. Cantero, T. Cao, M. D. M. Capeans Garrido, I. Caprini, M. Caprini, M. Capua, R. M. Carbone, R. Cardarelli, F. Cardillo, I. Carli, T. Carli, G. Carlino, B. T. Carlson, L. Carminati, R. M. D. Carney, S. Caron, E. Carquin, S. Carrá, G. D. Carrillo-Montoya, J. Carvalho, D. Casadei, M. P. Casado, M. Casolino, D. W. Casper, R. Castelijn, V. Castillo Gimenez, N. F. Castro, A. Catinaccio, J. R. Catmore, A. Cattai, J. Caudron, V. Cavaliere, E. Cavallaro, D. Cavalli, M. Cavalli-Sforza, V. Cavasinni, E. Celebi, F. Ceradini, L. Cerda Alberich, A. S. Cerqueira, A. Cerri, L. Cerrito, F. Cerutti, A. Cervelli, S. A. Cetin, A. Chafaq, D. Chakraborty, S. K. Chan, W. S. Chan, Y. L. Chan, P. Chang, J. D. Chapman, D. G. Charlton, C. C. Chau, C. A. Chavez Barajas, S. Che, S. Cheatham, A. Chegwidden, S. Chekanov, S. V. Chekulaev, G. A. Chelkov, M. A. Chelstowska, C. Chen, H. Chen, S. Chen, S. Chen, X. Chen, Y. Chen, H. C. Cheng, H. J. Cheng, A. Cheplakov, E. Cheremushkina, R. Cherkaoui El Moursli, V. Chernyatin, E. Cheu, L. Chevalier, V. Chiarella, G. Chiarelli, G. Chiodini, A. S. Chisholm, A. Chitan, Y. H. Chiu, M. V. Chizhov, K. Choi, A. R. Chomont, S. Chouridou, V. Christodoulou, D. Chromek-Burckhart, M. C. Chu, J. Chudoba, A. J. Chuinard, J. J. Chwastowski, L. Chytka, A. K. Ciftci, D. Cinca, V. Cindro, I. A. Cioara, C. Ciocca, A. Ciocio, F. Cirotto, Z. H. Citron, M. Citterio, M. Ciubancan, A. Clark, B. L. Clark, M. R. Clark, P. J. Clark, R. N. Clarke, C. Clement, Y. Coadou, M. Cobal, A. Coccaro, J. Cochran, L. Colasurdo, B. Cole, A. P. Colijn, J. Collot, T. Colombo, P. Conde Muiño, E. Coniavitis, S. H. Connell, I. A. Connelly, S. Constantinescu, G. Conti, F. Conventi, M. Cooke, A. M. Cooper-Sarkar, F. Cormier, K. J. R. Cormier, M. Corradi, F. Corriveau, A. Cortes-Gonzalez, G. Cortiana, G. Costa, M. J. Costa, D. Costanzo, G. Cottin, G. Cowan, B. E. Cox, K. Cranmer, S. J. Crawley, R. A. Creager, G. Cree, S. Crépé-Renaudin, F. Crescioli, W. A. Cribbs, M. Cristinziani, V. Croft, G. Crosetti, A. Cueto, T. Cuhadar Donszelmann, A. R. Cukierman, J. Cummings, M. Curatolo, J. Cúth, H. Czirr, P. Czodrowski, G. D’amen, S. D’Auria, L. D’eramo, M. D’Onofrio, M. J. Da Cunha Sargedas De Sousa, C. Da Via, W. Dabrowski, T. Dado, T. Dai, O. Dale, F. Dallaire, C. Dallapiccola, M. Dam, J. R. Dandoy, M. F. Daneri, N. P. Dang, A. C. Daniells, N. S. Dann, M. Danninger, M. Dano Hoffmann, V. Dao, G. Darbo, S. Darmora, J. Dassoulas, A. Dattagupta, T. Daubney, W. Davey, C. David, T. Davidek, M. Davies, D. R. Davis, P. Davison, E. Dawe, I. Dawson, K. De, R. de Asmundis, A. De Benedetti, S. De Castro, S. De Cecco, N. De Groot, P. de Jong, H. De la Torre, F. De Lorenzi, A. De Maria, D. De Pedis, A. De Salvo, U. De Sanctis, A. De Santo, K. De Vasconcelos Corga, J. B. De Vivie De Regie, W. J. Dearnaley, R. Debbe, C. Debenedetti, D. V. Dedovich, N. Dehghanian, I. Deigaard, M. Del Gaudio, J. Del Peso, T. Del Prete, D. Delgove, F. Deliot, C. M. Delitzsch, A. Dell’Acqua, L. Dell’Asta, M. Dell’Orso, M. Della Pietra, D. della Volpe, M. Delmastro, C. Delporte, P. A. Delsart, D. A. DeMarco, S. Demers, M. Demichev, A. Demilly, S. P. Denisov, D. Denysiuk, D. Derendarz, J. E. Derkaoui, F. Derue, P. Dervan, K. Desch, C. Deterre, K. Dette, M. R. Devesa, P. O. Deviveiros, A. Dewhurst, S. Dhaliwal, F. A. Di Bello, A. Di Ciaccio, L. Di Ciaccio, W. K. Di Clemente, C. Di Donato, A. Di Girolamo, B. Di Girolamo, B. Di Micco, R. Di Nardo, K. F. Di Petrillo, A. Di Simone, R. Di Sipio, D. Di Valentino, C. Diaconu, M. Diamond, F. A. Dias, M. A. Diaz, E. B. Diehl, J. Dietrich, S. Díez Cornell, A. Dimitrievska, J. Dingfelder, P. Dita, S. Dita, F. Dittus, F. Djama, T. Djobava, J. I. Djuvsland, M. A. B. do Vale, D. Dobos, M. Dobre, C. Doglioni, J. Dolejsi, Z. Dolezal, M. Donadelli, S. Donati, P. Dondero, J. Donini, J. Dopke, A. Doria, M. T. Dova, A. T. Doyle, E. Drechsler, M. Dris, Y. Du, J. Duarte-Campderros, A. Dubreuil, E. Duchovni, G. Duckeck, A. Ducourthial, O. A. Ducu, D. Duda, A. Dudarev, A. Chr. Dudder, E. M. Duffield, L. Duflot, M. Dührssen, M. Dumancic, A. E. Dumitriu, A. K. Duncan, M. Dunford, H. Duran Yildiz, M. Düren, A. Durglishvili, D. Duschinger, B. Dutta, M. Dyndal, B. S. Dziedzic, C. Eckardt, K. M. Ecker, R. C. Edgar, T. Eifert, G. Eigen, K. Einsweiler, T. Ekelof, M. El Kacimi, R. El Kosseifi, V. Ellajosyula, M. Ellert, S. Elles, F. Ellinghaus, A. A. Elliot, N. Ellis, J. Elmsheuser, M. Elsing, D. Emeliyanov, Y. Enari, O. C. Endner, J. S. Ennis, J. Erdmann, A. Ereditato, G. Ernis, M. Ernst, S. Errede, M. Escalier, C. Escobar, B. Esposito, O. Estrada Pastor, A. I. Etienvre, E. Etzion, H. Evans, A. Ezhilov, M. Ezzi, F. Fabbri, L. Fabbri, G. Facini, R. M. Fakhrutdinov, S. Falciano, R. J. Falla, J. Faltova, Y. Fang, M. Fanti, A. Farbin, A. Farilla, C. Farina, E. M. Farina, T. Farooque, S. Farrell, S. M. Farrington, P. Farthouat, F. Fassi, P. Fassnacht, D. Fassouliotis, M. Faucci Giannelli, A. Favareto, W. J. Fawcett, L. Fayard, O. L. Fedin, W. Fedorko, S. Feigl, L. Feligioni, C. Feng, E. J. Feng, H. Feng, M. J. Fenton, A. B. Fenyuk, L. Feremenga, P. Fernandez Martinez, S. Fernandez Perez, J. Ferrando, A. Ferrari, P. Ferrari, R. Ferrari, D. E. Ferreira de Lima, A. Ferrer, D. Ferrere, C. Ferretti, F. Fiedler, A. Filipčič, M. Filipuzzi, F. Filthaut, M. Fincke-Keeler, K. D. Finelli, M. C. N. Fiolhais, L. Fiorini, A. Fischer, C. Fischer, J. Fischer, W. C. Fisher, N. Flaschel, I. Fleck, P. Fleischmann, R. R. M. Fletcher, T. Flick, B. M. Flierl, L. R. Flores Castillo, M. J. Flowerdew, G. T. Forcolin, A. Formica, F. A. Förster, A. Forti, A. G. Foster, D. Fournier, H. Fox, S. Fracchia, P. Francavilla, M. Franchini, S. Franchino, D. Francis, L. Franconi, M. Franklin, M. Frate, M. Fraternali, D. Freeborn, S. M. Fressard-Batraneanu, B. Freund, D. Froidevaux, J. A. Frost, C. Fukunaga, T. Fusayasu, J. Fuster, C. Gabaldon, O. Gabizon, A. Gabrielli, A. Gabrielli, G. P. Gach, S. Gadatsch, S. Gadomski, G. Gagliardi, L. G. Gagnon, C. Galea, B. Galhardo, E. J. Gallas, B. J. Gallop, P. Gallus, G. Galster, K. K. Gan, S. Ganguly, Y. Gao, Y. S. Gao, F. M. Garay Walls, C. García, J. E. García Navarro, M. Garcia-Sciveres, R. W. Gardner, N. Garelli, V. Garonne, A. Gascon Bravo, K. Gasnikova, C. Gatti, A. Gaudiello, G. Gaudio, I. L. Gavrilenko, C. Gay, G. Gaycken, E. N. Gazis, C. N. P. Gee, J. Geisen, M. Geisen, M. P. Geisler, K. Gellerstedt, C. Gemme, M. H. Genest, C. Geng, S. Gentile, C. Gentsos, S. George, D. Gerbaudo, A. Gershon, G. Geßner, S. Ghasemi, M. Ghneimat, B. Giacobbe, S. Giagu, P. Giannetti, S. M. Gibson, M. Gignac, M. Gilchriese, D. Gillberg, G. Gilles, D. M. Gingrich, N. Giokaris, M. P. Giordani, F. M. Giorgi, P. F. Giraud, P. Giromini, D. Giugni, F. Giuli, C. Giuliani, M. Giulini, B. K. Gjelsten, S. Gkaitatzis, I. Gkialas, E. L. Gkougkousis, P. Gkountoumis, L. K. Gladilin, C. Glasman, J. Glatzer, P. C. F. Glaysher, A. Glazov, M. Goblirsch-Kolb, J. Godlewski, S. Goldfarb, T. Golling, D. Golubkov, A. Gomes, R. Gonçalo, R. Goncalves Gama, J. Goncalves Pinto Firmino Da Costa, G. Gonella, L. Gonella, A. Gongadze, S. González de la Hoz, S. Gonzalez-Sevilla, L. Goossens, P. A. Gorbounov, H. A. Gordon, I. Gorelov, B. Gorini, E. Gorini, A. Gorišek, A. T. Goshaw, C. Gössling, M. I. Gostkin, C. A. Gottardo, C. R. Goudet, D. Goujdami, A. G. Goussiou, N. Govender, E. Gozani, L. Graber, I. Grabowska-Bold, P. O. J. Gradin, J. Gramling, E. Gramstad, S. Grancagnolo, V. Gratchev, P. M. Gravila, C. Gray, H. M. Gray, Z. D. Greenwood, C. Grefe, K. Gregersen, I. M. Gregor, P. Grenier, K. Grevtsov, J. Griffiths, A. A. Grillo, K. Grimm, S. Grinstein, Ph. Gris, J.-F. Grivaz, S. Groh, E. Gross, J. Grosse-Knetter, G. C. Grossi, Z. J. Grout, A. Grummer, L. Guan, W. Guan, J. Guenther, F. Guescini, D. Guest, O. Gueta, B. Gui, E. Guido, T. Guillemin, S. Guindon, U. Gul, C. Gumpert, J. Guo, W. Guo, Y. Guo, R. Gupta, S. Gupta, G. Gustavino, P. Gutierrez, N. G. Gutierrez Ortiz, C. Gutschow, C. Guyot, M. P. Guzik, C. Gwenlan, C. B. Gwilliam, A. Haas, C. Haber, H. K. Hadavand, N. Haddad, A. Hadef, S. Hageböck, M. Hagihara, H. Hakobyan, M. Haleem, J. Haley, G. Halladjian, G. D. Hallewell, K. Hamacher, P. Hamal, K. Hamano, A. Hamilton, G. N. Hamity, P. G. Hamnett, L. Han, S. Han, K. Hanagaki, K. Hanawa, M. Hance, B. Haney, P. Hanke, J. B. Hansen, J. D. Hansen, M. C. Hansen, P. H. Hansen, K. Hara, A. S. Hard, T. Harenberg, F. Hariri, S. Harkusha, R. D. Harrington, P. F. Harrison, N. M. Hartmann, M. Hasegawa, Y. Hasegawa, A. Hasib, S. Hassani, S. Haug, R. Hauser, L. Hauswald, L. B. Havener, M. Havranek, C. M. Hawkes, R. J. Hawkings, D. Hayakawa, D. Hayden, C. P. Hays, J. M. Hays, H. S. Hayward, S. J. Haywood, S. J. Head, T. Heck, V. Hedberg, L. Heelan, K. K. Heidegger, S. Heim, T. Heim, B. Heinemann, J. J. Heinrich, L. Heinrich, C. Heinz, J. Hejbal, L. Helary, A. Held, S. Hellman, C. Helsens, R. C. W. Henderson, Y. Heng, S. Henkelmann, A. M. Henriques Correia, S. Henrot-Versille, G. H. Herbert, H. Herde, V. Herget, Y. Hernández Jiménez, H. Herr, G. Herten, R. Hertenberger, L. Hervas, T. C. Herwig, G. G. Hesketh, N. P. Hessey, J. W. Hetherly, S. Higashino, E. Higón-Rodriguez, E. Hill, J. C. Hill, K. H. Hiller, S. J. Hillier, M. Hils, I. Hinchliffe, M. Hirose, D. Hirschbuehl, B. Hiti, O. Hladik, X. Hoad, J. Hobbs, N. Hod, M. C. Hodgkinson, P. Hodgson, A. Hoecker, M. R. Hoeferkamp, F. Hoenig, D. Hohn, T. R. Holmes, M. Homann, S. Honda, T. Honda, T. M. Hong, B. H. Hooberman, W. H. Hopkins, Y. Horii, A. J. Horton, J.-Y. Hostachy, S. Hou, A. Hoummada, J. Howarth, J. Hoya, M. Hrabovsky, J. Hrdinka, I. Hristova, J. Hrivnac, T. Hryn’ova, A. Hrynevich, P. J. Hsu, S.-C. Hsu, Q. Hu, S. Hu, Y. Huang, Z. Hubacek, F. Hubaut, F. Huegging, T. B. Huffman, E. W. Hughes, G. Hughes, M. Huhtinen, P. Huo, N. Huseynov, J. Huston, J. Huth, G. Iacobucci, G. Iakovidis, I. Ibragimov, L. Iconomidou-Fayard, Z. Idrissi, P. Iengo, O. Igonkina, T. Iizawa, Y. Ikegami, M. Ikeno, Y. Ilchenko, D. Iliadis, N. Ilic, G. Introzzi, P. Ioannou, M. Iodice, K. Iordanidou, V. Ippolito, M. F. Isacson, N. Ishijima, M. Ishino, M. Ishitsuka, C. Issever, S. Istin, F. Ito, J. M. Iturbe Ponce, R. Iuppa, H. Iwasaki, J. M. Izen, V. Izzo, S. Jabbar, P. Jackson, R. M. Jacobs, V. Jain, K. B. Jakobi, K. Jakobs, S. Jakobsen, T. Jakoubek, D. O. Jamin, D. K. Jana, R. Jansky, J. Janssen, M. Janus, P. A. Janus, G. Jarlskog, N. Javadov, T. Javůrek, M. Javurkova, F. Jeanneau, L. Jeanty, J. Jejelava, A. Jelinskas, P. Jenni, C. Jeske, S. Jézéquel, H. Ji, J. Jia, H. Jiang, Y. Jiang, Z. Jiang, S. Jiggins, J. Jimenez Pena, S. Jin, A. Jinaru, O. Jinnouchi, H. Jivan, P. Johansson, K. A. Johns, C. A. Johnson, W. J. Johnson, K. Jon-And, R. W. L. Jones, S. D. Jones, S. Jones, T. J. Jones, J. Jongmanns, P. M. Jorge, J. Jovicevic, X. Ju, A. Juste Rozas, M. K. Köhler, A. Kaczmarska, M. Kado, H. Kagan, M. Kagan, S. J. Kahn, T. Kaji, E. Kajomovitz, C. W. Kalderon, A. Kaluza, S. Kama, A. Kamenshchikov, N. Kanaya, L. Kanjir, V. A. Kantserov, J. Kanzaki, B. Kaplan, L. S. Kaplan, D. Kar, K. Karakostas, N. Karastathis, M. J. Kareem, E. Karentzos, S. N. Karpov, Z. M. Karpova, K. Karthik, V. Kartvelishvili, A. N. Karyukhin, K. Kasahara, L. Kashif, R. D. Kass, A. Kastanas, Y. Kataoka, C. Kato, A. Katre, J. Katzy, K. Kawade, K. Kawagoe, T. Kawamoto, G. Kawamura, E. F. Kay, V. F. Kazanin, R. Keeler, R. Kehoe, J. S. Keller, J. J. Kempster, J Kendrick, H. Keoshkerian, O. Kepka, B. P. Kerševan, S. Kersten, R. A. Keyes, M. Khader, F. Khalil-zada, A. Khanov, A. G. Kharlamov, T. Kharlamova, A. Khodinov, T. J. Khoo, V. Khovanskiy, E. Khramov, J. Khubua, S. Kido, C. R. Kilby, H. Y. Kim, S. H. Kim, Y. K. Kim, N. Kimura, O. M. Kind, B. T. King, D. Kirchmeier, J. Kirk, A. E. Kiryunin, T. Kishimoto, D. Kisielewska, V. Kitali, K. Kiuchi, O. Kivernyk, E. Kladiva, T. Klapdor-Kleingrothaus, M. H. Klein, M. Klein, U. Klein, K. Kleinknecht, P. Klimek, A. Klimentov, R. Klingenberg, T. Klingl, T. Klioutchnikova, E.-E. Kluge, P. Kluit, S. Kluth, E. Kneringer, E. B. F. G. Knoops, A. Knue, A. Kobayashi, D. Kobayashi, T. Kobayashi, M. Kobel, M. Kocian, P. Kodys, T. Koffas, E. Koffeman, N. M. Köhler, T. Koi, M. Kolb, I. Koletsou, A. A. Komar, Y. Komori, T. Kondo, N. Kondrashova, K. Köneke, A. C. König, T. Kono, R. Konoplich, N. Konstantinidis, R. Kopeliansky, S. Koperny, A. K. Kopp, K. Korcyl, K. Kordas, A. Korn, A. A. Korol, I. Korolkov, E. V. Korolkova, O. Kortner, S. Kortner, T. Kosek, V. V. Kostyukhin, A. Kotwal, A. Koulouris, A. Kourkoumeli-Charalampidi, C. Kourkoumelis, E. Kourlitis, V. Kouskoura, A. B. Kowalewska, R. Kowalewski, T. Z. Kowalski, C. Kozakai, W. Kozanecki, A. S. Kozhin, V. A. Kramarenko, G. Kramberger, D. Krasnopevtsev, M. W. Krasny, A. Krasznahorkay, D. Krauss, J. A. Kremer, J. Kretzschmar, K. Kreutzfeldt, P. Krieger, K. Krizka, K. Kroeninger, H. Kroha, J. Kroll, J. Kroll, J. Kroseberg, J. Krstic, U. Kruchonak, H. Krüger, N. Krumnack, M. C. Kruse, T. Kubota, H. Kucuk, S. Kuday, J. T. Kuechler, S. Kuehn, A. Kugel, F. Kuger, T. Kuhl, V. Kukhtin, R. Kukla, Y. Kulchitsky, S. Kuleshov, Y. P. Kulinich, M. Kuna, T. Kunigo, A. Kupco, T. Kupfer, O. Kuprash, H. Kurashige, L. L. Kurchaninov, Y. A. Kurochkin, M. G. Kurth, V. Kus, E. S. Kuwertz, M. Kuze, J. Kvita, T. Kwan, D. Kyriazopoulos, A. La Rosa, J. L. La Rosa Navarro, L. La Rotonda, C. Lacasta, F. Lacava, J. Lacey, H. Lacker, D. Lacour, E. Ladygin, R. Lafaye, B. Laforge, T. Lagouri, S. Lai, S. Lammers, W. Lampl, E. Lançon, U. Landgraf, M. P. J. Landon, M. C. Lanfermann, V. S. Lang, J. C. Lange, R. J. Langenberg, A. J. Lankford, F. Lanni, K. Lantzsch, A. Lanza, A. Lapertosa, S. Laplace, J. F. Laporte, T. Lari, F. Lasagni Manghi, M. Lassnig, P. Laurelli, W. Lavrijsen, A. T. Law, P. Laycock, T. Lazovich, M. Lazzaroni, B. Le, O. Le Dortz, E. Le Guirriec, E. P. Le Quilleuc, M. LeBlanc, T. LeCompte, F. Ledroit-Guillon, C. A. Lee, G. R. Lee, S. C. Lee, L. Lee, B. Lefebvre, G. Lefebvre, M. Lefebvre, F. Legger, C. Leggett, A. Lehan, G. Lehmann Miotto, X. Lei, W. A. Leight, M. A. L. Leite, R. Leitner, D. Lellouch, B. Lemmer, K. J. C. Leney, T. Lenz, B. Lenzi, R. Leone, S. Leone, C. Leonidopoulos, G. Lerner, C. Leroy, A. A. J. Lesage, C. G. Lester, M. Levchenko, J. Levêque, D. Levin, L. J. Levinson, M. Levy, D. Lewis, B. Li, C. Li, H. Li, L. Li, Q. Li, S. Li, X. Li, Y. Li, Z. Liang, B. Liberti, A. Liblong, K. Lie, J. Liebal, W. Liebig, A. Limosani, S. C. Lin, T. H. Lin, B. E. Lindquist, A. E. Lionti, E. Lipeles, A. Lipniacka, M. Lisovyi, T. M. Liss, A. Lister, A. M. Litke, B. Liu, H. Liu, H. Liu, J. K. K. Liu, J. Liu, J. B. Liu, K. Liu, L. Liu, M. Liu, Y. L. Liu, Y. Liu, M. Livan, A. Lleres, J. Llorente Merino, S. L. Lloyd, C. Y. Lo, F. Lo Sterzo, E. M. Lobodzinska, P. Loch, F. K. Loebinger, A. Loesle, K. M. Loew, A. Loginov, T. Lohse, K. Lohwasser, M. Lokajicek, B. A. Long, J. D. Long, R. E. Long, L. Longo, K. A. Looper, J. A. Lopez, D. Lopez Mateos, I. Lopez Paz, A. Lopez Solis, J. Lorenz, N. Lorenzo Martinez, M. Losada, P. J. Lösel, X. Lou, A. Lounis, J. Love, P. A. Love, H. Lu, N. Lu, Y. J. Lu, H. J. Lubatti, C. Luci, A. Lucotte, C. Luedtke, F. Luehring, W. Lukas, L. Luminari, O. Lundberg, B. Lund-Jensen, P. M. Luzi, D. Lynn, R. Lysak, E. Lytken, V. Lyubushkin, H. Ma, L. L. Ma, Y. Ma, G. Maccarrone, A. Macchiolo, C. M. Macdonald, B. Maček, J. Machado Miguens, D. Madaffari, R. Madar, W. F. Mader, A. Madsen, J. Maeda, S. Maeland, T. Maeno, A. S. Maevskiy, E. Magradze, J. Mahlstedt, C. Maiani, C. Maidantchik, A. A. Maier, T. Maier, A. Maio, O. Majersky, S. Majewski, Y. Makida, N. Makovec, B. Malaescu, Pa. Malecki, V. P. Maleev, F. Malek, U. Mallik, D. Malon, C. Malone, S. Maltezos, S. Malyukov, J. Mamuzic, G. Mancini, L. Mandelli, I. Mandić, J. Maneira, L. Manhaes de Andrade Filho, J. Manjarres Ramos, A. Mann, A. Manousos, B. Mansoulie, J. D. Mansour, R. Mantifel, M. Mantoani, S. Manzoni, L. Mapelli, G. Marceca, L. March, L. Marchese, G. Marchiori, M. Marcisovsky, M. Marjanovic, D. E. Marley, F. Marroquim, S. P. Marsden, Z. Marshall, M. U. F Martensson, S. Marti-Garcia, C. B. Martin, T. A. Martin, V. J. Martin, B. Martin dit Latour, M. Martinez, V. I. Martinez Outschoorn, S. Martin-Haugh, V. S. Martoiu, A. C. Martyniuk, A. Marzin, L. Masetti, T. Mashimo, R. Mashinistov, J. Masik, A. L. Maslennikov, L. Massa, P. Mastrandrea, A. Mastroberardino, T. Masubuchi, P. Mättig, J. Maurer, S. J. Maxfield, D. A. Maximov, R. Mazini, I. Maznas, S. M. Mazza, N. C. Mc Fadden, G. Mc Goldrick, S. P. Mc Kee, A. McCarn, R. L. McCarthy, T. G. McCarthy, L. I. McClymont, E. F. McDonald, J. A. Mcfayden, G. Mchedlidze, S. J. McMahon, P. C. McNamara, R. A. McPherson, S. Meehan, T. J. Megy, S. Mehlhase, A. Mehta, T. Meideck, K. Meier, B. Meirose, D. Melini, B. R. Mellado Garcia, J. D. Mellenthin, M. Melo, F. Meloni, S. B. Menary, L. Meng, X. T. Meng, A. Mengarelli, S. Menke, E. Meoni, S. Mergelmeyer, P. Mermod, L. Merola, C. Meroni, F. S. Merritt, A. Messina, J. Metcalfe, A. S. Mete, C. Meyer, J.-P. Meyer, J. Meyer, H. Meyer Zu Theenhausen, F. Miano, R. P. Middleton, S. Miglioranzi, L. Mijović, G. Mikenberg, M. Mikestikova, M. Mikuž, M. Milesi, A. Milic, D. W. Miller, C. Mills, A. Milov, D. A. Milstead, A. A. Minaenko, Y. Minami, I. A. Minashvili, A. I. Mincer, B. Mindur, M. Mineev, Y. Minegishi, Y. Ming, L. M. Mir, K. P. Mistry, T. Mitani, J. Mitrevski, V. A. Mitsou, A. Miucci, P. S. Miyagawa, A. Mizukami, J. U. Mjörnmark, T. Mkrtchyan, M. Mlynarikova, T. Moa, K. Mochizuki, P. Mogg, S. Mohapatra, S. Molander, R. Moles-Valls, R. Monden, M. C. Mondragon, K. Mönig, J. Monk, E. Monnier, A. Montalbano, J. Montejo Berlingen, F. Monticelli, S. Monzani, R. W. Moore, N. Morange, D. Moreno, M. Moreno Llácer, P. Morettini, S. Morgenstern, D. Mori, T. Mori, M. Morii, M. Morinaga, V. Morisbak, A. K. Morley, G. Mornacchi, J. D. Morris, L. Morvaj, P. Moschovakos, M. Mosidze, H. J. Moss, J. Moss, K. Motohashi, R. Mount, E. Mountricha, E. J. W. Moyse, S. Muanza, R. D. Mudd, F. Mueller, J. Mueller, R. S. P. Mueller, D. Muenstermann, P. Mullen, G. A. Mullier, F. J. Munoz Sanchez, W. J. Murray, H. Musheghyan, M. Muškinja, A. G. Myagkov, M. Myska, B. P. Nachman, O. Nackenhorst, K. Nagai, R. Nagai, K. Nagano, Y. Nagasaka, K. Nagata, M. Nagel, E. Nagy, A. M. Nairz, Y. Nakahama, K. Nakamura, T. Nakamura, I. Nakano, R. F. Naranjo Garcia, R. Narayan, D. I. Narrias Villar, I. Naryshkin, T. Naumann, G. Navarro, R. Nayyar, H. A. Neal, P. Yu. Nechaeva, T. J. Neep, A. Negri, M. Negrini, S. Nektarijevic, C. Nellist, A. Nelson, M. E. Nelson, S. Nemecek, P. Nemethy, M. Nessi, M. S. Neubauer, M. Neumann, P. R. Newman, T. Y. Ng, T. Nguyen Manh, R. B. Nickerson, R. Nicolaidou, J. Nielsen, V. Nikolaenko, I. Nikolic-Audit, K. Nikolopoulos, J. K. Nilsen, P. Nilsson, Y. Ninomiya, A. Nisati, N. Nishu, R. Nisius, I. Nitsche, T. Nitta, T. Nobe, Y. Noguchi, M. Nomachi, I. Nomidis, M. A. Nomura, T. Nooney, M. Nordberg, N. Norjoharuddeen, O. Novgorodova, S. Nowak, M. Nozaki, L. Nozka, K. Ntekas, E. Nurse, F. Nuti, K. O’connor, D. C. O’Neil, A. A. O’Rourke, V. O’Shea, F. G. Oakham, H. Oberlack, T. Obermann, J. Ocariz, A. Ochi, I. Ochoa, J. P. Ochoa-Ricoux, S. Oda, S. Odaka, H. Ogren, A. Oh, S. H. Oh, C. C. Ohm, H. Ohman, H. Oide, H. Okawa, Y. Okumura, T. Okuyama, A. Olariu, L. F. Oleiro Seabra, S. A. Olivares Pino, D. Oliveira Damazio, A. Olszewski, J. Olszowska, A. Onofre, K. Onogi, P. U. E. Onyisi, M. J. Oreglia, Y. Oren, D. Orestano, N. Orlando, R. S. Orr, B. Osculati, R. Ospanov, G. Otero y Garzon, H. Otono, M. Ouchrif, F. Ould-Saada, A. Ouraou, K. P. Oussoren, Q. Ouyang, M. Owen, R. E. Owen, V. E. Ozcan, N. Ozturk, K. Pachal, A. Pacheco Pages, L. Pacheco Rodriguez, C. Padilla Aranda, S. Pagan Griso, M. Paganini, F. Paige, G. Palacino, S. Palazzo, S. Palestini, M. Palka, D. Pallin, E. St. Panagiotopoulou, I. Panagoulias, C. E. Pandini, J. G. Panduro Vazquez, P. Pani, S. Panitkin, D. Pantea, L. Paolozzi, Th. D. Papadopoulou, K. Papageorgiou, A. Paramonov, D. Paredes Hernandez, A. J. Parker, M. A. Parker, K. A. Parker, F. Parodi, J. A. Parsons, U. Parzefall, V. R. Pascuzzi, J. M. Pasner, E. Pasqualucci, S. Passaggio, Fr. Pastore, S. Pataraia, J. R. Pater, T. Pauly, B. Pearson, S. Pedraza Lopez, R. Pedro, S. V. Peleganchuk, O. Penc, C. Peng, H. Peng, J. Penwell, B. S. Peralva, M. M. Perego, D. V. Perepelitsa, F. Peri, L. Perini, H. Pernegger, S. Perrella, R. Peschke, V. D. Peshekhonov, K. Peters, R. F. Y. Peters, B. A. Petersen, T. C. Petersen, E. Petit, A. Petridis, C. Petridou, P. Petroff, E. Petrolo, M. Petrov, F. Petrucci, N. E. Pettersson, A. Peyaud, R. Pezoa, F. H. Phillips, P. W. Phillips, G. Piacquadio, E. Pianori, A. Picazio, E. Piccaro, M. A. Pickering, R. H. Pickles, R. Piegaia, J. E. Pilcher, A. D. Pilkington, A. W. J. Pin, M. Pinamonti, J. L. Pinfold, H. Pirumov, M. Pitt, L. Plazak, M.-A. Pleier, V. Pleskot, E. Plotnikova, D. Pluth, P. Podberezko, R. Poettgen, R. Poggi, L. Poggioli, D. Pohl, G. Polesello, A. Poley, A. Policicchio, R. Polifka, A. Polini, C. S. Pollard, V. Polychronakos, K. Pommès, D. Ponomarenko, L. Pontecorvo, B. G. Pope, G. A. Popeneciu, A. Poppleton, S. Pospisil, K. Potamianos, I. N. Potrap, C. J. Potter, G. Poulard, T. Poulsen, J. Poveda, M. E. Pozo Astigarraga, P. Pralavorio, A. Pranko, S. Prell, D. Price, L. E. Price, M. Primavera, S. Prince, N. Proklova, K. Prokofiev, F. Prokoshin, S. Protopopescu, J. Proudfoot, M. Przybycien, A. Puri, P. Puzo, J. Qian, G. Qin, Y. Qin, A. Quadt, M. Queitsch-Maitland, D. Quilty, S. Raddum, V. Radeka, V. Radescu, S. K. Radhakrishnan, P. Radloff, P. Rados, F. Ragusa, G. Rahal, J. A. Raine, S. Rajagopalan, C. Rangel-Smith, T. Rashid, S. Raspopov, M. G. Ratti, D. M. Rauch, F. Rauscher, S. Rave, I. Ravinovich, J. H. Rawling, M. Raymond, A. L. Read, N. P. Readioff, M. Reale, D. M. Rebuzzi, A. Redelbach, G. Redlinger, R. Reece, R. G. Reed, K. Reeves, L. Rehnisch, J. Reichert, A. Reiss, C. Rembser, H. Ren, M. Rescigno, S. Resconi, E. D. Resseguie, S. Rettie, E. Reynolds, O. L. Rezanova, P. Reznicek, R. Rezvani, R. Richter, S. Richter, E. Richter-Was, O. Ricken, M. Ridel, P. Rieck, C. J. Riegel, J. Rieger, O. Rifki, M. Rijssenbeek, A. Rimoldi, M. Rimoldi, L. Rinaldi, G. Ripellino, B. Ristić, E. Ritsch, I. Riu, F. Rizatdinova, E. Rizvi, C. Rizzi, R. T. Roberts, S. H. Robertson, A. Robichaud-Veronneau, D. Robinson, J. E. M. Robinson, A. Robson, E. Rocco, C. Roda, Y. Rodina, S. Rodriguez Bosca, A. Rodriguez Perez, D. Rodriguez Rodriguez, S. Roe, C. S. Rogan, O. Røhne, J. Roloff, A. Romaniouk, M. Romano, S. M. Romano Saez, E. Romero Adam, N. Rompotis, M. Ronzani, L. Roos, S. Rosati, K. Rosbach, P. Rose, N.-A. Rosien, E. Rossi, L. P. Rossi, J. H. N. Rosten, R. Rosten, M. Rotaru, I. Roth, J. Rothberg, D. Rousseau, A. Rozanov, Y. Rozen, X. Ruan, F. Rubbo, F. Rühr, A. Ruiz-Martinez, Z. Rurikova, N. A. Rusakovich, H. L. Russell, J. P. Rutherfoord, N. Ruthmann, Y. F. Ryabov, M. Rybar, G. Rybkin, S. Ryu, A. Ryzhov, G. F. Rzehorz, A. F. Saavedra, G. Sabato, S. Sacerdoti, H. F.-W. Sadrozinski, R. Sadykov, F. Safai Tehrani, P. Saha, M. Sahinsoy, M. Saimpert, M. Saito, T. Saito, H. Sakamoto, Y. Sakurai, G. Salamanna, J. E. Salazar Loyola, D. Salek, P. H. Sales De Bruin, D. Salihagic, A. Salnikov, J. Salt, D. Salvatore, F. Salvatore, A. Salvucci, A. Salzburger, D. Sammel, D. Sampsonidis, D. Sampsonidou, J. Sánchez, V. Sanchez Martinez, A. Sanchez Pineda, H. Sandaker, R. L. Sandbach, C. O. Sander, M. Sandhoff, C. Sandoval, D. P. C. Sankey, M. Sannino, A. Sansoni, C. Santoni, R. Santonico, H. Santos, I. Santoyo Castillo, A. Sapronov, J. G. Saraiva, B. Sarrazin, O. Sasaki, K. Sato, E. Sauvan, G. Savage, P. Savard, N. Savic, C. Sawyer, L. Sawyer, J. Saxon, C. Sbarra, A. Sbrizzi, T. Scanlon, D. A. Scannicchio, M. Scarcella, V. Scarfone, J. Schaarschmidt, P. Schacht, B. M. Schachtner, D. Schaefer, L. Schaefer, R. Schaefer, J. Schaeffer, S. Schaepe, S. Schaetzel, U. Schäfer, A. C. Schaffer, D. Schaile, R. D. Schamberger, V. Scharf, V. A. Schegelsky, D. Scheirich, M. Schernau, C. Schiavi, S. Schier, L. K. Schildgen, C. Schillo, M. Schioppa, S. Schlenker, K. R. Schmidt-Sommerfeld, K. Schmieden, C. Schmitt, S. Schmitt, S. Schmitz, U. Schnoor, L. Schoeffel, A. Schoening, B. D. Schoenrock, E. Schopf, M. Schott, J. F. P. Schouwenberg, J. Schovancova, S. Schramm, N. Schuh, A. Schulte, M. J. Schultens, H.-C. Schultz-Coulon, H. Schulz, M. Schumacher, B. A. Schumm, Ph. Schune, A. Schwartzman, T. A. Schwarz, H. Schweiger, Ph. Schwemling, R. Schwienhorst, J. Schwindling, A. Sciandra, G. Sciolla, F. Scuri, F. Scutti, J. Searcy, P. Seema, S. C. Seidel, A. Seiden, J. M. Seixas, G. Sekhniaidze, K. Sekhon, S. J. Sekula, N. Semprini-Cesari, S. Senkin, C. Serfon, L. Serin, L. Serkin, M. Sessa, R. Seuster, H. Severini, T. Sfiligoj, F. Sforza, A. Sfyrla, E. Shabalina, N. W. Shaikh, L. Y. Shan, R. Shang, J. T. Shank, M. Shapiro, P. B. Shatalov, K. Shaw, S. M. Shaw, A. Shcherbakova, C. Y. Shehu, Y. Shen, N. Sherafati, P. Sherwood, L. Shi, S. Shimizu, C. O. Shimmin, M. Shimojima, I. P. J. Shipsey, S. Shirabe, M. Shiyakova, J. Shlomi, A. Shmeleva, D. Shoaleh Saadi, M. J. Shochet, S. Shojaii, D. R. Shope, S. Shrestha, E. Shulga, M. A. Shupe, P. Sicho, A. M. Sickles, P. E. Sidebo, E. Sideras Haddad, O. Sidiropoulou, A. Sidoti, F. Siegert, Dj. Sijacki, J. Silva, S. B. Silverstein, V. Simak, Lj. Simic, S. Simion, E. Simioni, B. Simmons, M. Simon, P. Sinervo, N. B. Sinev, M. Sioli, G. Siragusa, I. Siral, S. Yu. Sivoklokov, J. Sjölin, M. B. Skinner, P. Skubic, M. Slater, T. Slavicek, M. Slawinska, K. Sliwa, R. Slovak, V. Smakhtin, B. H. Smart, J. Smiesko, N. Smirnov, S. Yu. Smirnov, Y. Smirnov, L. N. Smirnova, O. Smirnova, J. W. Smith, M. N. K. Smith, R. W. Smith, M. Smizanska, K. Smolek, A. A. Snesarev, I. M. Snyder, S. Snyder, R. Sobie, F. Socher, A. Soffer, D. A. Soh, G. Sokhrannyi, C. A. Solans Sanchez, M. Solar, E. Yu. Soldatov, U. Soldevila, A. A. Solodkov, A. Soloshenko, O. V. Solovyanov, V. Solovyev, P. Sommer, H. Son, A. Sopczak, D. Sosa, C. L. Sotiropoulou, R. Soualah, A. M. Soukharev, D. South, B. C. Sowden, S. Spagnolo, M. Spalla, M. Spangenberg, F. Spanò, D. Sperlich, F. Spettel, T. M. Spieker, R. Spighi, G. Spigo, L. A. Spiller, M. Spousta, R. D. St. Denis, A. Stabile, R. Stamen, S. Stamm, E. Stanecka, R. W. Stanek, C. Stanescu, M. M. Stanitzki, B. S. Stapf, S. Stapnes, E. A. Starchenko, G. H. Stark, J. Stark, S. H Stark, P. Staroba, P. Starovoitov, S. Stärz, R. Staszewski, P. Steinberg, B. Stelzer, H. J. Stelzer, O. Stelzer-Chilton, H. Stenzel, G. A. Stewart, M. C. Stockton, M. Stoebe, G. Stoicea, P. Stolte, S. Stonjek, A. R. Stradling, A. Straessner, M. E. Stramaglia, J. Strandberg, S. Strandberg, M. Strauss, P. Strizenec, R. Ströhmer, D. M. Strom, R. Stroynowski, A. Strubig, S. A. Stucci, B. Stugu, N. A. Styles, D. Su, J. Su, S. Suchek, Y. Sugaya, M. Suk, V. V. Sulin, D. M. S. Sultan, S. Sultansoy, T. Sumida, S. Sun, X. Sun, K. Suruliz, C. J. E. Suster, M. R. Sutton, S. Suzuki, M. Svatos, M. Swiatlowski, S. P. Swift, I. Sykora, T. Sykora, D. Ta, K. Tackmann, J. Taenzer, A. Taffard, R. Tafirout, N. Taiblum, H. Takai, R. Takashima, E. H. Takasugi, T. Takeshita, Y. Takubo, M. Talby, A. A. Talyshev, J. Tanaka, M. Tanaka, R. Tanaka, S. Tanaka, R. Tanioka, B. B. Tannenwald, S. Tapia Araya, S. Tapprogge, S. Tarem, G. F. Tartarelli, P. Tas, M. Tasevsky, T. Tashiro, E. Tassi, A. Tavares Delgado, Y. Tayalati, A. C. Taylor, G. N. Taylor, P. T. E. Taylor, W. Taylor, P. Teixeira-Dias, D. Temple, H. Ten Kate, P. K. Teng, J. J. Teoh, F. Tepel, S. Terada, K. Terashi, J. Terron, S. Terzo, M. Testa, R. J. Teuscher, T. Theveneaux-Pelzer, J. P. Thomas, J. Thomas-Wilsker, P. D. Thompson, A. S. Thompson, L. A. Thomsen, E. Thomson, M. J. Tibbetts, R. E. Ticse Torres, V. O. Tikhomirov, Yu. A. Tikhonov, S. Timoshenko, P. Tipton, S. Tisserant, K. Todome, S. Todorova-Nova, J. Tojo, S. Tokár, K. Tokushuku, E. Tolley, L. Tomlinson, M. Tomoto, L. Tompkins, K. Toms, B. Tong, P. Tornambe, E. Torrence, H. Torres, E. Torró Pastor, J. Toth, F. Touchard, D. R. Tovey, C. J. Treado, T. Trefzger, F. Tresoldi, A. Tricoli, I. M. Trigger, S. Trincaz-Duvoid, M. F. Tripiana, W. Trischuk, B. Trocmé, A. Trofymov, C. Troncon, M. Trottier-McDonald, M. Trovatelli, L. Truong, M. Trzebinski, A. Trzupek, K. W. Tsang, J. C.-L. Tseng, P. V. Tsiareshka, G. Tsipolitis, N. Tsirintanis, S. Tsiskaridze, V. Tsiskaridze, E. G. Tskhadadze, K. M. Tsui, I. I. Tsukerman, V. Tsulaia, S. Tsuno, D. Tsybychev, Y. Tu, A. Tudorache, V. Tudorache, T. T. Tulbure, A. N. Tuna, S. A. Tupputi, S. Turchikhin, D. Turgeman, I. Turk Cakir, R. Turra, P. M. Tuts, G. Ucchielli, I. Ueda, M. Ughetto, F. Ukegawa, G. Unal, A. Undrus, G. Unel, F. C. Ungaro, Y. Unno, C. Unverdorben, J. Urban, P. Urquijo, P. Urrejola, G. Usai, J. Usui, L. Vacavant, V. Vacek, B. Vachon, A. Vaidya, C. Valderanis, E. Valdes Santurio, S. Valentinetti, A. Valero, L. Valéry, S. Valkar, A. Vallier, J. A. Valls Ferrer, W. Van Den Wollenberg, H. van der Graaf, P. van Gemmeren, J. Van Nieuwkoop, I. van Vulpen, M. C. van Woerden, M. Vanadia, W. Vandelli, A. Vaniachine, P. Vankov, G. Vardanyan, R. Vari, E. W. Varnes, C. Varni, T. Varol, D. Varouchas, A. Vartapetian, K. E. Varvell, J. G. Vasquez, G. A. Vasquez, F. Vazeille, T. Vazquez Schroeder, J. Veatch, V. Veeraraghavan, L. M. Veloce, F. Veloso, S. Veneziano, A. Ventura, M. Venturi, N. Venturi, A. Venturini, V. Vercesi, M. Verducci, W. Verkerke, A. T. Vermeulen, J. C. Vermeulen, M. C. Vetterli, N. Viaux Maira, O. Viazlo, I. Vichou, T. Vickey, O. E. Vickey Boeriu, G. H. A. Viehhauser, S. Viel, L. Vigani, M. Villa, M. Villaplana Perez, E. Vilucchi, M. G. Vincter, V. B. Vinogradov, A. Vishwakarma, C. Vittori, I. Vivarelli, S. Vlachos, M. Vlasak, M. Vogel, P. Vokac, G. Volpi, H. von der Schmitt, E. von Toerne, V. Vorobel, K. Vorobev, M. Vos, R. Voss, J. H. Vossebeld, N. Vranjes, M. Vranjes Milosavljevic, V. Vrba, M. Vreeswijk, R. Vuillermet, I. Vukotic, P. Wagner, W. Wagner, J. Wagner-Kuhr, H. Wahlberg, S. Wahrmund, J. Wakabayashi, J. Walder, R. Walker, W. Walkowiak, V. Wallangen, C. Wang, C. Wang, F. Wang, H. Wang, H. Wang, J. Wang, J. Wang, Q. Wang, R. Wang, S. M. Wang, T. Wang, W. Wang, W. Wang, Z. Wang, C. Wanotayaroj, A. Warburton, C. P. Ward, D. R. Wardrope, A. Washbrook, P. M. Watkins, A. T. Watson, M. F. Watson, G. Watts, S. Watts, B. M. Waugh, A. F. Webb, S. Webb, M. S. Weber, S. W. Weber, S. A. Weber, J. S. Webster, A. R. Weidberg, B. Weinert, J. Weingarten, M. Weirich, C. Weiser, H. Weits, P. S. Wells, T. Wenaus, T. Wengler, S. Wenig, N. Wermes, M. D. Werner, P. Werner, M. Wessels, K. Whalen, N. L. Whallon, A. M. Wharton, A. S. White, A. White, M. J. White, R. White, D. Whiteson, B. W. Whitmore, F. J. Wickens, W. Wiedenmann, M. Wielers, C. Wiglesworth, L. A. M. Wiik-Fuchs, A. Wildauer, F. Wilk, H. G. Wilkens, H. H. Williams, S. Williams, C. Willis, S. Willocq, J. A. Wilson, I. Wingerter-Seez, E. Winkels, F. Winklmeier, O. J. Winston, B. T. Winter, M. Wittgen, M. Wobisch, T. M. H. Wolf, R. Wolff, M. W. Wolter, H. Wolters, V. W. S. Wong, S. D. Worm, B. K. Wosiek, J. Wotschack, K. W. Wozniak, M. Wu, S. L. Wu, X. Wu, Y. Wu, T. R. Wyatt, B. M. Wynne, S. Xella, Z. Xi, L. Xia, D. Xu, L. Xu, T. Xu, B. Yabsley, S. Yacoob, D. Yamaguchi, Y. Yamaguchi, A. Yamamoto, S. Yamamoto, T. Yamanaka, M. Yamatani, K. Yamauchi, Y. Yamazaki, Z. Yan, H. Yang, H. Yang, Y. Yang, Z. Yang, W.-M. Yao, Y. C. Yap, Y. Yasu, E. Yatsenko, K. H. Yau Wong, J. Ye, S. Ye, I. Yeletskikh, E. Yigitbasi, E. Yildirim, K. Yorita, K. Yoshihara, C. Young, C. J. S. Young, J. Yu, J. Yu, S. P. Y. Yuen, I. Yusuff, B. Zabinski, G. Zacharis, R. Zaidan, A. M. Zaitsev, N. Zakharchuk, J. Zalieckas, A. Zaman, S. Zambito, D. Zanzi, C. Zeitnitz, G. Zemaityte, A. Zemla, J. C. Zeng, Q. Zeng, O. Zenin, T. Ženiš, D. Zerwas, D. Zhang, F. Zhang, G. Zhang, H. Zhang, J. Zhang, L. Zhang, L. Zhang, M. Zhang, P. Zhang, R. Zhang, R. Zhang, X. Zhang, Y. Zhang, Z. Zhang, X. Zhao, Y. Zhao, Z. Zhao, A. Zhemchugov, B. Zhou, C. Zhou, L. Zhou, M. Zhou, M. Zhou, N. Zhou, C. G. Zhu, H. Zhu, J. Zhu, Y. Zhu, X. Zhuang, K. Zhukov, A. Zibell, D. Zieminska, N. I. Zimine, C. Zimmermann, S. Zimmermann, Z. Zinonos, M. Zinser, M. Ziolkowski, L. Živković, G. Zobernig, A. Zoccoli, R. Zou, M. zur Nedden, L. Zwalinski, ATLAS Collaboration

**Affiliations:** 1 Department of Physics, University of Adelaide, Adelaide, Australia; 2 Physics Department, SUNY Albany, Albany, NY USA; 3 Department of Physics, University of Alberta, Edmonton, AB Canada; 4 Department of Physics, Ankara University, Ankara, Turkey; 5 Istanbul Aydin University, Istanbul, Turkey; 6 Division of Physics, TOBB University of Economics and Technology, Ankara, Turkey; 7 LAPP, CNRS/IN2P3 and Université Savoie Mont Blanc, Annecy-le-Vieux, France; 8 High Energy Physics Division, Argonne National Laboratory, Argonne, IL USA; 9 Department of Physics, University of Arizona, Tucson, AZ USA; 10 Department of Physics, The University of Texas at Arlington, Arlington, TX USA; 11 Physics Department, National and Kapodistrian University of Athens, Athens, Greece; 12 Physics Department, National Technical University of Athens, Zografou, Greece; 13 Department of Physics, The University of Texas at Austin, Austin, TX USA; 14 Institute of Physics, Azerbaijan Academy of Sciences, Baku, Azerbaijan; 15 Institut de Física d’Altes Energies (IFAE), The Barcelona Institute of Science and Technology, Barcelona, Spain; 16 Institute of Physics, University of Belgrade, Belgrade, Serbia; 17 Department for Physics and Technology, University of Bergen, Bergen, Norway; 18 Physics Division, Lawrence Berkeley National Laboratory, University of California, Berkeley, CA USA; 19 Department of Physics, Humboldt University, Berlin, Germany; 20 Albert Einstein Center for Fundamental Physics, Laboratory for High Energy Physics, University of Bern, Bern, Switzerland; 21 School of Physics and Astronomy, University of Birmingham, Birmingham, UK; 22 Department of Physics, Bogazici University, Istanbul, Turkey; 23 Department of Physics Engineering, Gaziantep University, Gaziantep, Turkey; 24 Faculty of Engineering and Natural Sciences, Istanbul Bilgi University, Istanbul, Turkey; 25 Faculty of Engineering and Natural Sciences, Bahcesehir University, Istanbul, Turkey; 26 Centro de Investigaciones, Universidad Antonio Narino, Bogotá, Colombia; 27 INFN Sezione di Bologna, Bologna, Italy; 28 Dipartimento di Fisica e Astronomia, Università di Bologna, Bologna, Italy; 29 Physikalisches Institut, University of Bonn, Bonn, Germany; 30 Department of Physics, Boston University, Boston, MA USA; 31 Department of Physics, Brandeis University, Waltham, MA USA; 32 Universidade Federal do Rio De Janeiro COPPE/EE/IF, Rio de Janeiro, Brazil; 33 Electrical Circuits Department, Federal University of Juiz de Fora (UFJF), Juiz de Fora, Brazil; 34 Federal University of Sao Joao del Rei (UFSJ), Sao Joao del Rei, Brazil; 35 Instituto de Fisica, Universidade de Sao Paulo, São Paulo, Brazil; 36 Physics Department, Brookhaven National Laboratory, Upton, NY USA; 37 Transilvania University of Brasov, Brasov, Romania; 38 Horia Hulubei National Institute of Physics and Nuclear Engineering, Bucharest, Romania; 39 Department of Physics, Alexandru Ioan Cuza University of Iasi, Iasi, Romania; 40 Physics Department, National Institute for Research and Development of Isotopic and Molecular Technologies, Cluj-Napoca, Romania; 41 University Politehnica Bucharest, Bucharest, Romania; 42 West University in Timisoara, Timisoara, Romania; 43 Departamento de Física, Universidad de Buenos Aires, Buenos Aires, Argentina; 44 Cavendish Laboratory, University of Cambridge, Cambridge, UK; 45 Department of Physics, Carleton University, Ottawa, ON Canada; 46 CERN, Geneva, Switzerland; 47 Enrico Fermi Institute, University of Chicago, Chicago, IL USA; 48 Departamento de Física, Pontificia Universidad Católica de Chile, Santiago, Chile; 49 Departamento de Física, Universidad Técnica Federico Santa María, Valparaiso, Chile; 50 Institute of High Energy Physics, Chinese Academy of Sciences, Beijing, China; 51 Department of Physics, Nanjing University, Nanjing, Jiangsu China; 52 Physics Department, Tsinghua University, Beijing, 100084 China; 53 Department of Modern Physics and State Key Laboratory of Particle Detection and Electronics, University of Science and Technology of China, Hefei, Anhui China; 54 School of Physics, Shandong University, Jinan, Shandong China; 55 Department of Physics and Astronomy, Key Laboratory for Particle Physics, Astrophysics and Cosmology, Ministry of Education, Shanghai Key Laboratory for Particle Physics and Cosmology, Shanghai Jiao Tong University, Shanghai (also at PKU-CHEP), Shanghai, China; 56 Université Clermont Auvergne, CNRS/IN2P3, LPC, Clermont-Ferrand, France; 57 Nevis Laboratory, Columbia University, Irvington, NY USA; 58 Niels Bohr Institute, University of Copenhagen, Copenhagen, Denmark; 59 INFN Gruppo Collegato di Cosenza, Laboratori Nazionali di Frascati, Frascati, Italy; 60 Dipartimento di Fisica, Università della Calabria, Rende, Italy; 61 Faculty of Physics and Applied Computer Science, AGH University of Science and Technology, Kraków, Poland; 62 Marian Smoluchowski Institute of Physics, Jagiellonian University, Kraków, Poland; 63 Institute of Nuclear Physics, Polish Academy of Sciences, Kraków, Poland; 64 Physics Department, Southern Methodist University, Dallas, TX USA; 65 Physics Department, University of Texas at Dallas, Richardson, TX USA; 66 DESY, Hamburg and Zeuthen, Germany; 67 Lehrstuhl für Experimentelle Physik IV, Technische Universität Dortmund, Dortmund, Germany; 68 Institut für Kern- und Teilchenphysik, Technische Universität Dresden, Dresden, Germany; 69 Department of Physics, Duke University, Durham, NC USA; 70 SUPA-School of Physics and Astronomy, University of Edinburgh, Edinburgh, UK; 71 INFN e Laboratori Nazionali di Frascati, Frascati, Italy; 72 Fakultät für Mathematik und Physik, Albert-Ludwigs-Universität, Freiburg, Germany; 73 Departement de Physique Nucleaire et Corpusculaire, Université de Genève, Geneva, Switzerland; 74 INFN Sezione di Genova, Genoa, Italy; 75 Dipartimento di Fisica, Università di Genova, Genoa, Italy; 76 E. Andronikashvili Institute of Physics, Iv. Javakhishvili Tbilisi State University, Tbilisi, Georgia; 77 High Energy Physics Institute, Tbilisi State University, Tbilisi, Georgia; 78 II Physikalisches Institut, Justus-Liebig-Universität Giessen, Giessen, Germany; 79 SUPA-School of Physics and Astronomy, University of Glasgow, Glasgow, UK; 80 II Physikalisches Institut, Georg-August-Universität, Göttingen, Germany; 81 Laboratoire de Physique Subatomique et de Cosmologie, Université Grenoble-Alpes, CNRS/IN2P3, Grenoble, France; 82 Laboratory for Particle Physics and Cosmology, Harvard University, Cambridge, MA USA; 83 Kirchhoff-Institut für Physik, Ruprecht-Karls-Universität Heidelberg, Heidelberg, Germany; 84 Physikalisches Institut, Ruprecht-Karls-Universität Heidelberg, Heidelberg, Germany; 85 ZITI Institut für technische Informatik, Ruprecht-Karls-Universität Heidelberg, Mannheim, Germany; 86 Faculty of Applied Information Science, Hiroshima Institute of Technology, Hiroshima, Japan; 87 Department of Physics, The Chinese University of Hong Kong, Shatin, NT Hong Kong; 88 Department of Physics, The University of Hong Kong, Hong Kong, China; 89 Department of Physics, Institute for Advanced Study, The Hong Kong University of Science and Technology, Clear Water Bay, Kowloon, Hong Kong, China; 90 Department of Physics, National Tsing Hua University, Hsinchu, Taiwan; 91 Department of Physics, Indiana University, Bloomington, IN USA; 92 Institut für Astro- und Teilchenphysik, Leopold-Franzens-Universität, Innsbruck, Austria; 93 University of Iowa, Iowa City, IA USA; 94 Department of Physics and Astronomy, Iowa State University, Ames, IA USA; 95 Joint Institute for Nuclear Research, JINR Dubna, Dubna, Russia; 96 KEK, High Energy Accelerator Research Organization, Tsukuba, Japan; 97 Graduate School of Science, Kobe University, Kobe, Japan; 98 Faculty of Science, Kyoto University, Kyoto, Japan; 99 Kyoto University of Education, Kyoto, Japan; 100 Research Center for Advanced Particle Physics and Department of Physics, Kyushu University, Fukuoka, Japan; 101 Instituto de Física La Plata, Universidad Nacional de La Plata and CONICET, La Plata, Argentina; 102 Physics Department, Lancaster University, Lancaster, UK; 103 INFN Sezione di Lecce, Lecce, Italy; 104 Dipartimento di Matematica e Fisica, Università del Salento, Lecce, Italy; 105 Oliver Lodge Laboratory, University of Liverpool, Liverpool, UK; 106 Department of Experimental Particle Physics, Jožef Stefan Institute and Department of Physics, University of Ljubljana, Ljubljana, Slovenia; 107 School of Physics and Astronomy, Queen Mary University of London, London, UK; 108 Department of Physics, Royal Holloway University of London, Surrey, UK; 109 Department of Physics and Astronomy, University College London, London, UK; 110 Louisiana Tech University, Ruston, LA USA; 111 Laboratoire de Physique Nucléaire et de Hautes Energies, UPMC and Université Paris-Diderot and CNRS/IN2P3, Paris, France; 112 Fysiska institutionen, Lunds universitet, Lund, Sweden; 113 Departamento de Fisica Teorica C-15, Universidad Autonoma de Madrid, Madrid, Spain; 114 Institut für Physik, Universität Mainz, Mainz, Germany; 115 School of Physics and Astronomy, University of Manchester, Manchester, UK; 116 CPPM, Aix-Marseille Université and CNRS/IN2P3, Marseille, France; 117 Department of Physics, University of Massachusetts, Amherst, MA USA; 118 Department of Physics, McGill University, Montreal, QC Canada; 119 School of Physics, University of Melbourne, Victoria, Australia; 120 Department of Physics, The University of Michigan, Ann Arbor, MI USA; 121 Department of Physics and Astronomy, Michigan State University, East Lansing, MI USA; 122 INFN Sezione di Milano, Milan, Italy; 123 Dipartimento di Fisica, Università di Milano, Milan, Italy; 124 B.I. Stepanov Institute of Physics, National Academy of Sciences of Belarus, Minsk, Republic of Belarus; 125 Research Institute for Nuclear Problems of Byelorussian State University, Minsk, Republic of Belarus; 126 Group of Particle Physics, University of Montreal, Montreal, QC Canada; 127 P.N. Lebedev Physical Institute of the Russian Academy of Sciences, Moscow, Russia; 128 Institute for Theoretical and Experimental Physics (ITEP), Moscow, Russia; 129 National Research Nuclear University MEPhI, Moscow, Russia; 130 D.V. Skobeltsyn Institute of Nuclear Physics, M.V. Lomonosov Moscow State University, Moscow, Russia; 131 Fakultät für Physik, Ludwig-Maximilians-Universität München, Munich, Germany; 132 Max-Planck-Institut für Physik (Werner-Heisenberg-Institut), Munich, Germany; 133 Nagasaki Institute of Applied Science, Nagasaki, Japan; 134 Graduate School of Science and Kobayashi-Maskawa Institute, Nagoya University, Nagoya, Japan; 135 INFN Sezione di Napoli, Naples, Italy; 136 Dipartimento di Fisica, Università di Napoli, Naples, Italy; 137 Department of Physics and Astronomy, University of New Mexico, Albuquerque, NM USA; 138 Institute for Mathematics, Astrophysics and Particle Physics, Radboud University Nijmegen/Nikhef, Nijmegen, The Netherlands; 139 Nikhef National Institute for Subatomic Physics, University of Amsterdam, Amsterdam, The Netherlands; 140 Department of Physics, Northern Illinois University, DeKalb, IL USA; 141 Budker Institute of Nuclear Physics, SB RAS, Novosibirsk, Russia; 142 Department of Physics, New York University, New York, NY USA; 143 Ohio State University, Columbus, OH USA; 144 Faculty of Science, Okayama University, Okayama, Japan; 145 Homer L. Dodge Department of Physics and Astronomy, University of Oklahoma, Norman, OK USA; 146 Department of Physics, Oklahoma State University, Stillwater, OK USA; 147 Palacký University, RCPTM, Olomouc, Czech Republic; 148 Center for High Energy Physics, University of Oregon, Eugene, OR USA; 149 LAL, Univ. Paris-Sud, CNRS/IN2P3, Université Paris-Saclay, Orsay, France; 150 Graduate School of Science, Osaka University, Osaka, Japan; 151 Department of Physics, University of Oslo, Oslo, Norway; 152 Department of Physics, Oxford University, Oxford, UK; 153 INFN Sezione di Pavia, Pavia, Italy; 154 Dipartimento di Fisica, Università di Pavia, Pavia, Italy; 155 Department of Physics, University of Pennsylvania, Philadelphia, PA USA; 156 National Research Centre “Kurchatov Institute” B.P. Konstantinov Petersburg Nuclear Physics Institute, St. Petersburg, Russia; 157 INFN Sezione di Pisa, Pisa, Italy; 158 Dipartimento di Fisica E. Fermi, Università di Pisa, Pisa, Italy; 159 Department of Physics and Astronomy, University of Pittsburgh, Pittsburgh, PA USA; 160 Laboratório de Instrumentação e Física Experimental de Partículas-LIP, Lisbon, Portugal; 161 Faculdade de Ciências, Universidade de Lisboa, Lisbon, Portugal; 162 Department of Physics, University of Coimbra, Coimbra, Portugal; 163 Centro de Física Nuclear da Universidade de Lisboa, Lisbon, Portugal; 164 Departamento de Fisica, Universidade do Minho, Braga, Portugal; 165 Departamento de Fisica Teorica y del Cosmos and CAFPE, Universidad de Granada, Granada, Spain; 166 Dep Fisica and CEFITEC of Faculdade de Ciencias e Tecnologia, Universidade Nova de Lisboa, Caparica, Portugal; 167 Institute of Physics, Academy of Sciences of the Czech Republic, Prague, Czech Republic; 168 Czech Technical University in Prague, Prague, Czech Republic; 169 Faculty of Mathematics and Physics, Charles University, Prague, Czech Republic; 170 State Research Center Institute for High Energy Physics (Protvino), NRC KI, Protvino, Russia; 171 Particle Physics Department, Rutherford Appleton Laboratory, Didcot, UK; 172 INFN Sezione di Roma, Rome, Italy; 173 Dipartimento di Fisica, Sapienza Università di Roma, Rome, Italy; 174 INFN Sezione di Roma Tor Vergata, Rome, Italy; 175 Dipartimento di Fisica, Università di Roma Tor Vergata, Rome, Italy; 176 INFN Sezione di Roma Tre, Rome, Italy; 177 Dipartimento di Matematica e Fisica, Università Roma Tre, Rome, Italy; 178 Faculté des Sciences Ain Chock, Réseau Universitaire de Physique des Hautes Energies-Université Hassan II, Casablanca, Morocco; 179 Centre National de l’Energie des Sciences Techniques Nucleaires, Rabat, Morocco; 180 Faculté des Sciences Semlalia, Université Cadi Ayyad, LPHEA-Marrakech, Marrakech, Morocco; 181 Faculté des Sciences, Université Mohamed Premier and LPTPM, Oujda, Morocco; 182 Faculté des Sciences, Université Mohammed V, Rabat, Morocco; 183 DSM/IRFU (Institut de Recherches sur les Lois Fondamentales de l’Univers), CEA Saclay (Commissariat à l’Energie Atomique et aux Energies Alternatives), Gif-sur-Yvette, France; 184 Santa Cruz Institute for Particle Physics, University of California Santa Cruz, Santa Cruz, CA USA; 185 Department of Physics, University of Washington, Seattle, WA USA; 186 Department of Physics and Astronomy, University of Sheffield, Sheffield, UK; 187 Department of Physics, Shinshu University, Nagano, Japan; 188 Department Physik, Universität Siegen, Siegen, Germany; 189 Department of Physics, Simon Fraser University, Burnaby, BC Canada; 190 SLAC National Accelerator Laboratory, Stanford, CA USA; 191 Faculty of Mathematics, Physics and Informatics, Comenius University, Bratislava, Slovak Republic; 192 Department of Subnuclear Physics, Institute of Experimental Physics of the Slovak Academy of Sciences, Kosice, Slovak Republic; 193 Department of Physics, University of Cape Town, Cape Town, South Africa; 194 Department of Physics, University of Johannesburg, Johannesburg, South Africa; 195 School of Physics, University of the Witwatersrand, Johannesburg, South Africa; 196 Department of Physics, Stockholm University, Stockholm, Sweden; 197 The Oskar Klein Centre, Stockholm, Sweden; 198 Physics Department, Royal Institute of Technology, Stockholm, Sweden; 199 Departments of Physics and Astronomy and Chemistry, Stony Brook University, Stony Brook, NY USA; 200 Department of Physics and Astronomy, University of Sussex, Brighton, UK; 201 School of Physics, University of Sydney, Sydney, Australia; 202 Institute of Physics, Academia Sinica, Taipei, Taiwan; 203 Department of Physics, Technion: Israel Institute of Technology, Haifa, Israel; 204 Raymond and Beverly Sackler School of Physics and Astronomy, Tel Aviv University, Tel Aviv, Israel; 205 Department of Physics, Aristotle University of Thessaloniki, Thessaloníki, Greece; 206 International Center for Elementary Particle Physics and Department of Physics, The University of Tokyo, Tokyo, Japan; 207 Graduate School of Science and Technology, Tokyo Metropolitan University, Tokyo, Japan; 208 Department of Physics, Tokyo Institute of Technology, Tokyo, Japan; 209 Tomsk State University, Tomsk, Russia; 210 Department of Physics, University of Toronto, Toronto, ON Canada; 211 INFN-TIFPA, Trento, Italy; 212 University of Trento, Trento, Italy; 213 TRIUMF, Vancouver, BC Canada; 214 Department of Physics and Astronomy, York University, Toronto, ON Canada; 215 Faculty of Pure and Applied Sciences, and Center for Integrated Research in Fundamental Science and Engineering, University of Tsukuba, Tsukuba, Japan; 216 Department of Physics and Astronomy, Tufts University, Medford, MA USA; 217 Department of Physics and Astronomy, University of California Irvine, Irvine, CA USA; 218 INFN Gruppo Collegato di Udine, Sezione di Trieste, Udine, Italy; 219 ICTP, Trieste, Italy; 220 Dipartimento di Chimica, Fisica e Ambiente, Università di Udine, Udine, Italy; 221 Department of Physics and Astronomy, University of Uppsala, Uppsala, Sweden; 222 Department of Physics, University of Illinois, Urbana, IL USA; 223 Instituto de Fisica Corpuscular (IFIC), Centro Mixto Universidad de Valencia-CSIC, Valencia, Spain; 224 Department of Physics, University of British Columbia, Vancouver, BC Canada; 225 Department of Physics and Astronomy, University of Victoria, Victoria, BC Canada; 226 Department of Physics, University of Warwick, Coventry, UK; 227 Waseda University, Tokyo, Japan; 228 Department of Particle Physics, The Weizmann Institute of Science, Rehovot, Israel; 229 Department of Physics, University of Wisconsin, Madison, WI USA; 230 Fakultät für Physik und Astronomie, Julius-Maximilians-Universität, Würzburg, Germany; 231 Fakultät für Mathematik und Naturwissenschaften, Fachgruppe Physik, Bergische Universität Wuppertal, Wuppertal, Germany; 232 Department of Physics, Yale University, New Haven, CT USA; 233 Yerevan Physics Institute, Yerevan, Armenia; 234 Centre de Calcul de l’Institut National de Physique Nucléaire et de Physique des Particules (IN2P3), Villeurbanne, France; 235 Academia Sinica Grid Computing, Institute of Physics, Academia Sinica, Taipei, Taiwan; 236 CERN, 1211 Geneva 23, Switzerland

## Abstract

Observables sensitive to the anomalous production of events containing hadronic jets and missing momentum in the plane transverse to the proton beams at the Large Hadron Collider are presented. The observables are defined as a ratio of cross sections, for events containing jets and large missing transverse momentum to events containing jets and a pair of charged leptons from the decay of a $Z/\gamma ^*$ boson. This definition minimises experimental and theoretical systematic uncertainties in the measurements. This ratio is measured differentially with respect to a number of kinematic properties of the hadronic system in two phase-space regions; one inclusive single-jet region and one region sensitive to vector-boson-fusion topologies. The data are found to be in agreement with the Standard Model predictions and used to constrain a variety of theoretical models for dark-matter production, including simplified models, effective field theory models, and invisible decays of the Higgs boson. The measurements use 3.2 fb$^{-1}$ of proton–proton collision data recorded by the ATLAS experiment at a centre-of-mass energy of 13 $\text {TeV}$ and are fully corrected for detector effects, meaning that the data can be used to constrain new-physics models beyond those shown in this paper.

## Introduction

The Standard Model of particle physics (SM) is an extremely successful theory, describing the fundamental building blocks of nature and the interactions between them. Despite its many successes, it is known that the SM does not provide a complete description: for example it does not explain the abundance of dark matter in our universe, known to exist from astrophysical observations [1–3]. One of the main aims of the physics programme at the Large Hadron Collider (LHC) [4] is to find evidence of new phenomena, either via directly searching for the signatures predicted by specific scenarios beyond the Standard Model (BSM) or, as is the case in this paper, by performing a more general search for deviations from SM predictions.

**Fig. 1:**
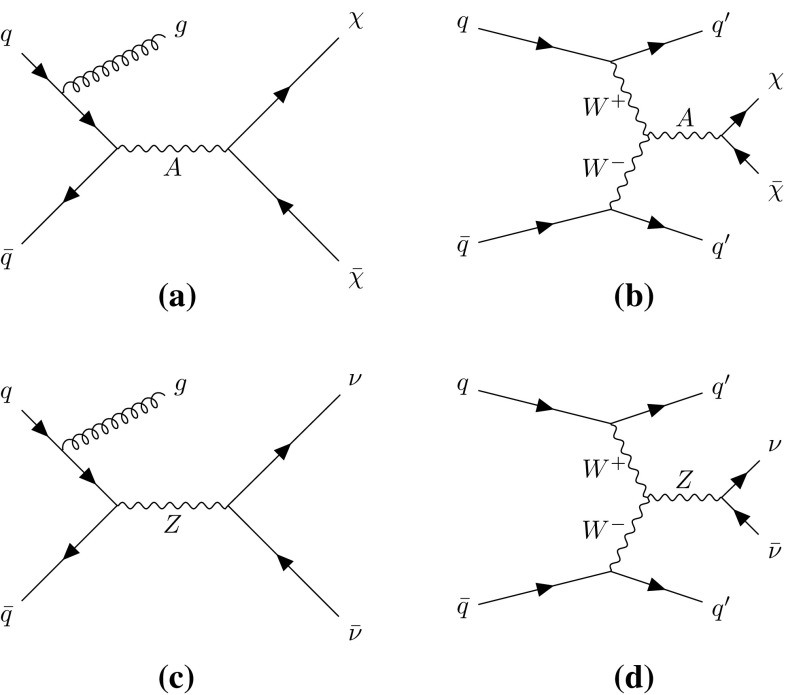
Example Feynman diagrams for WIMP $\chi$ pair production with mediator *A* produced **a** in association with one jet and **b** via vector-boson fusion. Example Feynman diagrams for the Standard Model background to **c** the process with one jet and **d** the vector-boson fusion process

New physics phenomena at the LHC may manifest themselves as events with jets of collimated, mostly hadronic, particles and a momentum imbalance in the plane transverse to the LHC beams, known as missing transverse momentum, $p_\mathrm {T}^\mathrm {miss}$. The $p_\mathrm {T}^\mathrm {miss}$ may indicate the presence of particles that do not interact via the strong or electromagnetic interactions and therefore cannot be directly detected in the LHC detectors. These particles are referred to as invisible. In particular, new-physics models predicting the existence of weakly interacting massive particles (WIMPs), dark-matter candidates that could be produced at the LHC, could lead to such a signature [5]. As an example, a Feynman diagram is shown in Fig. 1a, where a mediator, *A*, is produced in association with a gluon-initiated jet and decays to a WIMP pair ($\chi \bar{\chi }$). Limits have previously been placed in such models by comparing the number of events in $p_\mathrm {T}^\mathrm {miss}{} + \mathrm {jets}$ final states in LHC data with the number of background events expected to be seen in the detector (the detector level) [6, 7]. Another possible production mechanism for the experimental observation of weakly interacting BSM particles is vector-boson fusion (VBF) [8], as shown in Fig. 1b. This is a topology similar to that in the invisible decay of a VBF-produced Higgs boson [9–11], for which limits have previously been set [12, 13] using detector-level data. The dominant SM process leading to the same final states is the production of a $Z$ boson in association with jets, where the $Z$ boson decays to a pair of neutrinos. Example diagrams are shown in Fig. 1c, d.

This paper presents a measurement of differential observables that are sensitive to the anomalous production of events containing one or more hadronic jets with high transverse momentum, $p_{\text {T}}$, produced in association with a large $p_\mathrm {T}^\mathrm {miss}$. The measurements are performed using data corresponding to an integrated luminosity of 3.2 fb$^{-1}$ of proton–proton collisions at $\sqrt{s}=13$ $\text {TeV}$, collected by the ATLAS detector [14] in 2015. The observables are corrected for detector inefficiencies and resolutions and are presented at the particle level. They are constructed from a ratio of cross-sections,

$$\begin{aligned} R^{\mathrm {miss}} = \frac{\sigma _{\text {fid}} \left( p_\mathrm {T}^\mathrm {miss}{} + \mathrm {jets}{} \right) }{\sigma _{\text {fid}} \left( \ell ^{+}\ell ^{-} + \mathrm {jets}{} \right) }, \end{aligned}$$

defined in a fiducial phase space. The numerator is the fiducial cross-section for $p_\mathrm {T}^\mathrm {miss}{} + \mathrm {jets}$ events, which corresponds to the fiducial cross-section for inclusive $Z(\rightarrow \nu \bar{\nu })+$ jets production in the SM. The denominator is the fiducial cross-section for $\ell ^{+}\ell ^{-} + \mathrm {jets}$ events, where the unobserved system that produces the $p_\mathrm {T}^\mathrm {miss}$ in the numerator is replaced by an observed, opposite-sign, same-flavour pair of charged leptons consistent with originating from a $Z/\gamma ^*$ boson. The lepton pair can be either a pair of electrons or muons. The jet system is required to satisfy very similar selection criteria in both the $p_\mathrm {T}^\mathrm {miss}{} + \mathrm {jets}$ and $\ell ^{+}\ell ^{-} + \mathrm {jets}$ samples of events so as to significantly reduce experimental and theoretical uncertainties in the ratio measurement. The presence of BSM physics in the numerator would lead to a discrepancy between the measured ratio and that predicted by the SM.

The approach used in this paper allows for direct comparison of SM and BSM predictions at the particle level, without the need to simulate the effects of the ATLAS detector. This is computationally efficient and enables those without access to a precise simulation of the ATLAS detector to compare the data with predictions from alternative BSM models as they become available. Since each alternative BSM model may predict event signatures with different kinematic properties, the publication of the kinematic distributions enhances the usefulness and longevity of the data. Furthermore, future improvements in the predictions of the SM processes that contribute to the ratio can be compared to the particle-level data and limits in BSM models can be updated accordingly.

Particle-level measurements of SM processes are common in collider physics and have, on occasion, been used to set limits in BSM models (see e.g. [15]), although not to search for new physics in the $p_\mathrm {T}^\mathrm {miss}{} + \mathrm {jets}$ final state. Moreover, a measurement of the particle-level ratio allows the denominator to provide a constraint on the dominant SM process contributing to the $p_\mathrm {T}^\mathrm {miss}{} + \mathrm {jets}$ final state. Many sources of systematic uncertainty cancel in the ratio because the requirements on the hadronic system and the definition of the measured kinematic variables, determined from the hadronic system, are similar in the numerator $p_\mathrm {T}^\mathrm {miss}{} + \mathrm {jets}$ and denominator $\ell ^{+}\ell ^{-} + \mathrm {jets}$ events. This is made possible by treating the identified charged leptons in $\ell ^{+}\ell ^{-} + \mathrm {jets}$ events as invisible when calculating the $p_\mathrm {T}^\mathrm {miss}$. This cancellation occurs, for example, for phenomenological uncertainties in the prediction of initial-state parton radiation and experimental uncertainties in the jet reconstruction, energy scale and resolution.

The ratio measurements are presented in two phase-space regions: the $\ge 1 \, \mathrm {jet}$ region, containing at least one high-$p_{\text {T}}$ jet, and the VBF region, containing at least two high-$p_{\text {T}}$ jets, and satisfying additional selection criteria to enhance the VBF process. This ratio is measured as a function of a number of kinematic properties of the hadronic system of the event and the statistical and systematic correlations between the different distributions are determined. The data and correlation information are made publicly available.

The remainder of this paper is laid out as follows. The ATLAS detector and event reconstruction are described in Sect. 2. The fiducial regions defined by particle-level objects and event selections, together with the measured variables, are detailed in Sect. 3. The $p_\mathrm {T}^\mathrm {miss}{} + \mathrm {jets}$ and $\ell ^{+}\ell ^{-} + \mathrm {jets}$ event samples are selected as described in Sect. 4. Samples of events were produced with Monte Carlo event generators and are used to correct the data for detector effects, to estimate background and signal contributions, and to assign systematic uncertainties to the results. Details of these samples are given in Sect. 5. Predicted backgrounds, explained in Sect. 6, are subtracted from the selected data and the ratio is computed. A correction for detector effects is applied to the ratios, as described in Sect. 7, so that they are defined at particle level with the definitions from Sect. 3. Systematic uncertainties in the measurement and theoretical predictions are summarised in Sect. 8. The detector-corrected events in the electron and muon channels are combined to form particle-level ratios to $\ell ^{+}\ell ^{-} + \mathrm {jets}$ events, as described in Sect. 9. These are compared to the expected SM ratios and to the expected ratios including example BSM models in Sect. 10. The results are discussed in Sect. 11 and example limits are placed on BSM model parameters. Finally, conclusions are given in Sect. 12.

## ATLAS detector and event reconstruction

The ATLAS detector [14, 16, 17] is a multipurpose particle detector with a cylindrical geometry. ATLAS consists of layers of tracking detectors, calorimeters, and muon chambers. The inner detector (ID) covers the pseudorapidity range $|\eta | < 2.5$.^1^ The ID is immersed in a 2 T magnetic field and measures the trajectories and momenta of charged particles. The calorimeter covers the pseudorapidity range $|\eta | < 4.9$. Within $|\eta | <2.47$, the finely segmented electromagnetic calorimeter identifies electromagnetic showers and measures their energy and position, providing electron identification together with the ID. The muon spectrometer (MS) surrounds the calorimeters and provides muon identification and measurement in the region $|\eta | < 2.7$.

Jets are reconstructed from energy deposits in the calorimeters, using the anti-$k_t$ jet algorithm [18, 19], with a jet-radius parameter of 0.4. The measured jet $p_{\text {T}}$ is corrected [20] for the detector response and contributions to the jet energy from multiple proton–proton interactions (pileup). Jet quality selection criteria [21] are applied. Track-based variables are then used to suppress jets with $|\eta |<2.4$ and $p_{\text {T}} < 50$ $\text {GeV}$ by requiring that a significant fraction of the tracks associated with each jet must have an origin compatible with the primary vertex in the event, which further suppresses jets from pileup interactions.

A muon is reconstructed by matching a track (or track segment) reconstructed in the MS to a track reconstructed in the ID. Its momentum is calculated by combining the information from the two systems and correcting for energy deposited in the calorimeters. Quality requirements are applied using the *loose* working point as described in Ref. [22]. An electron is reconstructed from an energy deposit (cluster) in the electromagnetic calorimeter matched to a track in the ID. Its momentum is computed from the cluster energy and the direction of the track. Electrons are distinguished from other particles using several identification criteria that rely on the shapes of electromagnetic showers as well as tracking and track-to-cluster matching quantities. The output of a likelihood function taking these quantities as input, similar to that described in Ref. [23], and using the *loose* working point described therein, is used to identify electrons. Data-driven energy/momentum scale corrections [22] are applied to both reconstructed muons and electrons. Leptons are required to be associated with the primary vertex, defined as the vertex with the highest $\Sigma p_{\text {T}} ^2$ of its associated tracks, in order to suppress leptons originating from pileup and secondary decays. Hadronic decays of $\tau$ leptons ($\tau \rightarrow$ hadrons $+ \nu$) are predominantly characterised by the presence of one or three charged particles and possibly neutral pions. A multivariate boosted decision tree identification, based on calorimetric shower shape and track multiplicity of the $\tau$ candidates, is used to reject jets faking $\tau$ leptons. More details are given in Ref. [24], with the *loose* working point being used in this analysis.

The $p_\mathrm {T}^\mathrm {miss}$ is reconstructed as the magnitude of the negative vector sum of the transverse momenta of all detected particles, as described in Ref. [25]. The $p_\mathrm {T}^\mathrm {miss}$ calculation uses a soft term that is calculated using tracks within the ID which are not associated with jets or with leptons that are being treated as invisible particles. The momenta of calibrated jets with $p_{\text {T}} > 20$ $\text {GeV}$ are used.

Events in the numerator and the  denominator are selected by a trigger that requires $p_\mathrm {T}^\mathrm {miss}> 70$ $\text {GeV}$, as computed in the final stage of the two-level trigger system. Since the momenta from muons are not included in the $p_\mathrm {T}^\mathrm {miss}$ calculation in this trigger, the muons appear to the trigger as invisible particles and hence the trigger can also be used to select  events. This trigger is 100% efficient for the offline $p_\mathrm {T}^\mathrm {miss}> 200$ $\text {GeV}$ requirement used in the analysis. Events in the  denominator are selected by a single-electron trigger, with an efficiency ranging between 93% and more than 99% for electrons with $p_{\text {T}} >80$ $\text {GeV}$, depending on their pseudorapidity.

## Particle-level objects, event selections and measured variables

The detector-corrected data are presented in fiducial regions defined in this section. The definition of the measured variables is also given. The final state of an event is defined using all particles with $c\tau$ longer than 10 mm. Final-state particles that interact via the strong or electromagnetic interactions are referred to as visible particles, whereas those that interact via neither are referred to as invisible particles.

At particle level, the $\ell ^{+}\ell ^{-} + \mathrm {jets}$ events for the denominator of $R^{\mathrm {miss}}$ are required to have exactly one opposite-sign, same-flavour pair of prompt^2^ leptons: an $e^+e^-$ or $\mu ^+\mu ^-$ pair. The four-momenta of prompt photons within a cone of $\Delta R= \sqrt{(\Delta \eta )^2 + (\Delta \phi )^2} = 0.1$ around each lepton are added to the four-momenta of the leptons and then removed from the final state, as motivated in Ref. [26]. These so-called ‘dressed’ leptons are required to satisfy the kinematic criteria detailed below.

Both the numerator and denominator of $R^{\mathrm {miss}}$ are required to satisfy a number of phase-space-dependent criteria, summarised in Table 1. The fiducial phase-space definitions are motivated by the acceptance of the detector and the trigger [27], background reduction and, in the case of the VBF phase space, by the enhancement of the contribution from VBF processes. The $p_\mathrm {T}^\mathrm {miss}$ value is defined as the magnitude of the negative vector sum of the transverse momenta of all visible final-state particles with $|\eta |<4.9$, as this corresponds to the edge of the calorimeter. Muons with $|\eta | > 2.5$ are excluded as they contribute only negligibly to the calculation of $p_\mathrm {T}^\mathrm {miss}$ in this analysis, via a small energy deposition in the calorimeter. For the denominator, the $p_\mathrm {T}^\mathrm {miss}$ variable is modified: the selected dressed leptons are excluded from the vector sum, making the variable very similar between numerator and denominator. Jets are reconstructed with the anti-$k_t$ jet algorithm with jet radius parameter 0.4, excluding invisible particles and muons.

The event-level veto on (additional) leptons is applied to reduce the contribution from background processes. In particular, this requirement significantly reduces the background to $p_\mathrm {T}^\mathrm {miss}{} + \mathrm {jets}$ events from $W$ bosons produced in association with jets. The requirement on the difference in azimuthal angle between $p_\mathrm {T}^\mathrm {miss}$ and any of the leading four jets with $p_{\text {T}} > 30$ $\text {GeV}$, $\Delta \phi _{\mathrm {jet_{i},p_\mathrm {T}^\mathrm {miss}}}$ , suppresses backgrounds from multijet events, as is discussed in Sect. 6. For the denominator, the minimum $p_{\text {T}}$ requirement for the leading lepton is much larger than the subleading lepton as events with a large $p_\mathrm {T}^\mathrm {miss}$ tend to have one very high $p_{\text {T}}$ lepton. The subleading lepton $p_{\text {T}}$ can be much lower, in particular if it is in the direction opposite the decaying $Z$ boson. The leading lepton $p_{\text {T}}$ tends to be lower in $t\bar{t}$ events, motivating the choice to make an asymmetric requirement. The requirement on the dilepton invariant mass to be between 66 and 116 $\text {GeV}$ is implemented to minimise the contribution of the photon propagator and interference terms in the denominator, making it as similar as possible to the numerator.

In VBF, at least two jets are in the final state and, due to the colourless exchange, less hadronic activity in the rapidity space between the two jets is expected, which motivates the central-jet veto. The dijet invariant mass ($m_{\mathrm {jj}}$ ) requirement suppresses the contribution from diboson events where one boson decays hadronically.

**Table 1:** Definitions for the $\ge 1 \, \mathrm {jet}$ and VBF fiducial phase spaces. Here $m_{\mathrm {jj}}$ is the invariant mass of the two leading (in $p_{\text {T}}$ ) jets, $\Delta \phi _{\mathrm {jet_{i},p_\mathrm {T}^\mathrm {miss}}}$ is the difference in azimuthal angle between $p_\mathrm {T}^\mathrm {miss}$ and a jet axis. The lepton veto is applied to events in the numerator (denominator) of $R^{\mathrm {miss}}$ containing at least one (three) prompt lepton(s) or lepton(s) from $\tau$ decays. The selected leptons in the denominator are treated as invisible when calculating the $p_\mathrm {T}^\mathrm {miss}$ value. The central-jet veto is applied to any jets in the rapidity (*y*) space between the two leading jets. The dilepton invariant mass is denoted by $m_{\ell \ell }$

Numerator and denominator	$\ge 1 \, \mathrm {jet}$	VBF
$p_\mathrm {T}^\mathrm {miss}$	$> 200$ $\text {GeV}$	
(Additional) lepton veto	No $e,\mu$ with $p_{\text {T}} > 7$ $\text {GeV}$, $|\eta | < 2.5$	
Jet |*y*|	$< 4.4$	
Jet $p_{\text {T}}$	$> 25$ $\text {GeV}$	
$\Delta \phi _{\mathrm {jet_{i},p_\mathrm {T}^\mathrm {miss}}}$	$> 0.4$, for the four leading jets with $p_{\text {T}} > 30$ $\text {GeV}$	
Leading jet $p_{\text {T}}$	$> 120$ GeV	$> 80$ $\text {GeV}$
Subleading jet $p_{\text {T}}$	–	$> 50$ $\text {GeV}$
Leading jet $|\eta |$	$< 2.4$	–
$m_{\mathrm {jj}}$	–	$> 200$ $\text {GeV}$
Central-jet veto	–	No jets with $p_{\text {T}} > 25$ $\text {GeV}$

Denominator only	$\ge 1 \, \mathrm {jet}$ and VBF	
Leading lepton $p_{\text {T}}$	$> 80$ $\text {GeV}$	
Subleading lepton $p_{\text {T}}$	$> 7$ $\text {GeV}$	
Lepton $|\eta |$	$< 2.5$	
$m_{\ell \ell }$	66–116 $\text {GeV}$	
$\Delta R$ (jet, lepton)	$> 0.5$, otherwise jet is removed	

In order to increase the sensitivity to a range of targeted BSM scenarios, four differential measurements of $R^{\mathrm {miss}}$ are made with respect to: $p_\mathrm {T}^\mathrm {miss}$ in the $\ge 1 \, \mathrm {jet}$ and VBF phase spaces, as well as $m_{\mathrm {jj}}$ and $\Delta \phi _{\mathrm {jj}}$ in the VBF phase space, where $\Delta \phi _{\mathrm {jj}}$ is the difference in azimuthal angle between the two leading jets. Due to the larger mediator mass and higher energy scale of the interaction, many BSM signatures tend to have harder $p_\mathrm {T}^\mathrm {miss}$ distributions than the SM processes, meaning that sensitivity to these models is enhanced in the high-$p_\mathrm {T}^\mathrm {miss}$ region. Since the VBF process leads to events with a harder $m_{\mathrm {jj}}$ spectrum than processes involving the strong production of dijets, the high-$m_{\mathrm {jj}}$ region gives more discriminating power for VBF models. The expected $\Delta \phi _{\mathrm {jj}}$ distribution varies between different BSM theories and could therefore give additional sensitivity and possibly help to distinguish between models, should a signal be seen.

## Detector-level event selection

Events are required to contain a primary vertex with at least two associated tracks, each with $p_{\text {T}} > 400$ $\text {MeV}$. Events containing a jet with $p_{\text {T}} > 20$ $\text {GeV}$ not originating from a proton–proton interaction are rejected. Such jets are identified by jet quality selection criteria involving quantities such as the pulse shape of the energy depositions in the cells of the calorimeters, electromagnetic fraction in the calorimeter, calorimeter sampling fraction, or the fraction of energy coming from charged particles.

The kinematic selection criteria given in Table 1 are identically applied to detector-level objects, with an additional exclusion of electrons in the region $1.37< |\eta | < 1.52$, which corresponds to the calorimeter barrel–endcap transition region, and in the region $2.47< |\eta | < 2.5$, since electrons are identified only for $|\eta | <2.47$. All electrons, as well as muons used for the lepton veto, are required to be isolated from other particles. In both cases, the LooseTrackOnly isolation working points described in Refs. [22, 23] are used. A veto on events containing an identified hadronically decaying $\tau$ lepton, with the total $p_{\text {T}}$ of the visible decay products being greater than 20 $\text {GeV}$, is also applied to reduce the contribution from  events to $p_\mathrm {T}^\mathrm {miss}{} + \mathrm {jets}$ events. This veto is not applied at the particle level due to the complication of defining a hadronically decaying $\tau$ lepton in terms of stable final-state particles.

In this analysis, identified charged leptons are either vetoed or treated as invisible particles in the $p_\mathrm {T}^\mathrm {miss}$ calculation. In particular, for the $\ell ^{+}\ell ^{-} + \mathrm {jets}$ denominator, the measured momenta of selected electrons, muons, and jets close to muons which are consistent with being associated with final-state radiation photons clustered close to the muon ID track, are treated as invisible. A jet is considered to be consistent with a final-state photon if its transverse momentum is less than twice the transverse momentum of the associated muon and it has fewer than five associated ID tracks. This makes $p_\mathrm {T}^\mathrm {miss}$ very similar between numerator and denominator.

## Monte Carlo simulation

Events containing $Z$ and $W$ bosons (collectively termed *V*) were generated using Monte Carlo (MC) event generators. Samples contributing to inclusive $Z+$jets production ($Z \rightarrow \nu \bar{\nu }$ , $Z/\gamma ^* \rightarrow \ell ^{+}\ell ^{-}$ and diboson *ZV*, where the $Z$ decays to a $\nu \bar{\nu }$, $e^+e^-$ or $\mu ^+\mu ^-$ pair and *V* is a hadronically decaying $W$ or $Z$ boson) are used for the detector corrections. Samples of  (including *WV* where the $W$ decays leptonically and the *V* decays hadronically), top–antitop quark pairs, single-top-quark and leptonically decaying diboson (*WW*, *WZ*, *ZZ*) events are used to estimate backgrounds.

Events containing single $Z$ and $W$ bosons in association with jets were simulated using the Sherpa v2.2.0 event generator [28]. Matrix elements were calculated for up to two additional parton emissions at next-to-leading-order (NLO) accuracy and up to four additional parton emissions at leading-order (LO) accuracy using the Comix [29] and OpenLoops [30] matrix element generators and merged with the Sherpa parton shower [31], which is based on Catani–Seymour subtraction terms. The merging of multi-parton matrix elements with the parton shower is achieved using an improved CKKW matching procedure [32, 33], which is extended to NLO accuracy using the MEPS@NLO prescription [34]. The NNPDF3.0nnlo parton distribution function (PDF) set [35] was used in conjunction with the dedicated parton-shower tuning developed by the Sherpa authors. These $V+$jets samples were produced with a simplified scale-setting prescription in the multi-parton matrix elements to improve the event generation speed. A theory-based reweighting of the jet-multiplicity distribution is applied, derived from event generation with the strict scale prescription. The samples are normalised to a next-to-next-to-leading-order (NNLO) prediction [36]. The full set-up is described in detail in Ref. [37]. Electroweakly produced $V+$jets as well as diboson production were generated using Sherpa v2.1.1 in conjunction with the CT10nlo [38] PDF set and the dedicated parton-shower tuning developed by the Sherpa authors. The full set-up is described in detail in Ref. [39].

Alternative samples of events with $V+$jets simulated using MG5_aMC@NLO v2.2.2 [40] at LO and interfaced to the Pythia v8.186 [41] parton shower are used for cross-checks and for the determination of systematic uncertainties. The ATLAS A14 set of tuned parameters [42] is used together with the NNPDF3.0nlo PDF set. These samples are also normalised to the NNLO prediction.

Top–antitop pair production [43], as well as single-top-quark production in the *Wt* [44] and *s*-channels [45, 46], were generated using the Powheg-Box v2 [47–49] event generator with the CT10nlo PDF set for the matrix element calculations. Single-top *t*-channel events were generated using the Powheg-Box v1 event generator. Parton showering, hadronisation, and the underlying event were provided by Pythia v6.428 [50] using the CTEQ6L1 PDF set [51] and the Perugia 2012 (P2012) set of tuned parton-shower parameters [52]. The full set-up of these top-quark samples is described in detail in Ref. [53]. The top-pair samples are normalised to a calculation at NNLO accuracy including soft-gluon resummation at next-to-next-to-leading logarithmic (NNLL) accuracy [54]. The single-top samples are normalised using an NLO calculation including the resummation of soft gluon terms at NNLL accuracy [55–57].

WIMP simplified signal models were simulated using Powheg-Box v2 (r3049) using the model described in Ref. [58]. This model implements the production of WIMP pairs with *s*-channel spin-1 mediator exchange at NLO precision. Events were generated with the NNPDF3.0nlo PDF set with parton showering using Pythia v8.205 [59] with the A14 [42] parameter set. This model has a coupling $g_q$ of the SM quarks to the mediator, and a coupling $g_{\chi }$ of dark-matter particles to the mediator. Couplings were set to a constant value of $g_q = 0.25$ and $g_{\chi }=1$, as recommended in Ref. [60]. A grid of samples was produced for WIMP masses ranging from 1 $\text {GeV}$ to 1 $\text {TeV}$ and axial-vector mediator masses between 10 $\text {GeV}$ and 2 $\text {TeV}$. More details of the samples are given in Ref. [6].

In order to assess the sensitivity to invisible decays of the Higgs boson, $H \rightarrow ZZ \rightarrow 4\nu$ events were simulated using Powheg-Box v1 [61–63] with CT10 PDFs, and Pythia v8.165 simulating the parton shower, hadronisation and underlying event. The cross-sections and their uncertainties for Higgs boson production via vector-boson fusion, gluon–gluon fusion, and associated production are taken from Ref. [64].

In order to search for general signatures of Dirac-fermion dark-matter coupling to weak bosons, an implementation [65] of an effective field theory [8] (EFT) in FeynRules v2.3.1 [66] was used, with MadGraph5 v2.2.3 [40] used to simulate the hard interaction. This EFT includes ten possible dimension-five to dimension-seven operators with a range of possible Lorentz structures, including some with different charge-parity (*CP*) properties for the effective interaction between weak bosons and a dark matter candidate. This model was interfaced to Pythia v8.212 with the A14 parameter set and the NNPDF23LO [67] PDF to simulate the effects of parton showering, hadronisation and the underlying event.

All SM MC simulation samples were passed through GEANT4 [68, 69] for a full simulation [70] of the detector and are then reconstructed using the same analysis chain as the data. Scale factors are applied to the simulated events to correct for the small differences from data in the trigger, reconstruction, identification, isolation, and impact parameter efficiencies for leptons [22, 23]. Furthermore, the lepton and jet momentum scales and resolutions are adjusted to match the data. Additional proton–proton collisions in the same bunch crossing are overlaid. These are based on soft strong-interaction processes simulated with Pythia v8.186 using the MSTW2008lo PDF set [71] along with the A2 set of tuned parton-shower parameters [72]. The average number of proton–proton interactions per bunch crossing in this data set is 13.7.

## Backgrounds

The dominant background in the $p_\mathrm {T}^\mathrm {miss}{} + \mathrm {jets}$ numerator is from events containing a leptonically decaying $W$ boson produced in association with jets, which contain $p_\mathrm {T}^\mathrm {miss}$ associated with an invisible particle: in this case the neutrino in the $W$ decay. Such events would pass the veto on additional leptons if the charged lepton ($e, \mu$ or $\tau$) is not reconstructed or is outside the acceptance of the detector. This background includes contributions where the $W$ boson originates from a top-quark decay or diboson events. The top-quark decay contribution to the $W$ background amounts to approximately 18% (14%) in the $\ge 1 \, \mathrm {jet}$ (VBF) phase spaces. The three lepton decay channels of the $W$ background contribute approximately 18% ( ), 12% ( ) and 15% ( ) to the numerator. The size of the combined $W$ background is similar to the SM $Z \rightarrow \nu \bar{\nu }$ contribution to the numerator at low $p_\mathrm {T}^\mathrm {miss}$, becoming less important at high $p_\mathrm {T}^\mathrm {miss}$.

The contribution from this background is estimated using two $W$ control regions. A  ( ) control region is selected by requiring a muon (electron) that is isolated from other particles, with $p_{\text {T}} {} > 25$ $\text {GeV}$. The requirements on the jets, $p_\mathrm {T}^\mathrm {miss}$, and the veto on additional leptons are identical to those of the $p_\mathrm {T}^\mathrm {miss}{} + \mathrm {jets}$ signal region. In the  control region, the muon is treated as an invisible particle in the $p_\mathrm {T}^\mathrm {miss}$ calculation, in order to make the region as similar as possible to the signal region. This is because the signal region has a veto on reconstructed muons and so the muon is often not included in the $p_\mathrm {T}^\mathrm {miss}$ calculation. In the  control region, the energy of the electron is included in the $p_\mathrm {T}^\mathrm {miss}$ calculation, calibrated as a jet. This is because the electron is usually included in the signal region for  events, where the electron is generally inside the acceptance of the calorimeter, but is not identified, as a veto on identified electrons is applied in the signal region.  events, where the $\tau$ decay includes a muon (electron), are included in the  ( ) control regions so that the contribution of these events to the signal region is also included in this estimate.

The data in the  and  control regions are collected using the $p_\mathrm {T}^\mathrm {miss}$ and single-electron triggers discussed in Sect. 4 and are corrected for lepton inefficiencies on an event-by-event basis using $p_{\text {T}}$- and $\eta$-dependent lepton reconstruction, identification and isolation efficiencies, $\epsilon$, that were previously determined from data [22, 23]. The data in the  control region are also corrected for the single-electron trigger inefficiency. A small background contribution from multijet events in the control region is estimated using dedicated MC simulation and subtracted from the data. The efficiency- and multijet-corrected data are then used to predict the contribution from  and  events in the signal, which contains two types of events: those for which the lepton is inside the detector acceptance with $p_{\text {T}} {} > 7$ $\text {GeV}$ but does not pass the lepton reconstruction and identification criteria, and those with a lepton that is outside of the detector acceptance or has $p_{\text {T}} {} < 7$ $\text {GeV}$. The in-acceptance contribution is determined for each bin of a given distribution from the efficiency-corrected data in the control region by applying an additional weight of $\left( 1 - \epsilon \right)$ per event as well as correcting for the small difference in lepton fiducial acceptance between the control region and the signal region, using an acceptance-correction factor that is estimated using MC simulation. The out-of-acceptance contribution is obtained by extrapolating efficiency-corrected in-acceptance data using again acceptance corrections derived from simulation. As a cross-check, the $W$ background estimate is also determined using an alternative method, described in Ref. [6], where no efficiency weights are applied to data and the simulation is used to extrapolate from the control region to the signal region. Compatible results are found.

There is no specific  control region for hadronically decaying taus, as it is difficult to obtain a pure sample of  events in data. Instead, background predictions for  with hadronically decaying $\tau$ leptons are obtained by reweighting the simulated  events, in each bin of each distribution, by the ratio of efficiency-corrected data to simulation determined in the  or  control regions. The midpoint of the two predictions, obtained using the two control regions, is taken as the final  prediction and the difference between the midpoint and the two predictions is taken as a systematic uncertainty. This choice is made because a hadronically decaying $\tau$ lepton is often included in the $p_\mathrm {T}^\mathrm {miss}$ calculation, calibrated as a jet, which is similar to the  control region. However, the $\tau$ decay includes a neutrino, meaning that some part of it is invisible, which is similar to the  control region.

A much smaller background to the $p_\mathrm {T}^\mathrm {miss}{} + \mathrm {jets}$ events arises from multijet events in which one or more jets are mismeasured leading to a large measured $p_\mathrm {T}^\mathrm {miss}$. This implies that the $p_\mathrm {T}^\mathrm {miss}$ direction is likely to point towards one of the jets and so most of this background is removed by the $\Delta \phi _{\mathrm {jet_{i},p_\mathrm {T}^\mathrm {miss}}}$ requirement. The remaining background is estimated using a control region where at least one of the four leading jets satisfies the criterion $\Delta \phi _{\mathrm {jet_{i},p_\mathrm {T}^\mathrm {miss}}} < 0.1$. A large multijet data sample is obtained from events selected with single-jet triggers. These control events are required to be well measured, meaning that the $p_\mathrm {T}^\mathrm {miss}$ is low. In order to obtain a sample of events that pass the $p_\mathrm {T}^\mathrm {miss}$ selection, the jets in these events are smeared 25,000 times per multijet control event, according to the full jet response distribution. This sample is used to extrapolate between the control region and the signal region. The multijet background amounts to 2% in the first $p_\mathrm {T}^\mathrm {miss}$ bin, rapidly becoming negligible in the higher $p_\mathrm {T}^\mathrm {miss}$ bins. The small (0.5%) $Z/\gamma ^* \rightarrow \ell ^{+}\ell ^{-}$ background to the $p_\mathrm {T}^\mathrm {miss}{} + \mathrm {jets}$ events is estimated using MC simulation.

The background to $\ell ^{+}\ell ^{-} + \mathrm {jets}$ events is dominated by top–antitop quark pairs, with smaller contributions from diboson, single-top-quark, $W$ + jet and $Z\rightarrow \tau ^{+}\tau ^{-}$ events. These backgrounds are all estimated with MC simulation together with a control region that selects differently flavoured $\ell ^{+}\ell ^{-} + \mathrm {jets}$ events (an $e^{\pm }\mu ^{\mp }$ pair). All other selection criteria are the same. This control region removes the contribution from same-flavour $\ell ^{+}\ell ^{-} + \mathrm {jets}$ events but retains contributions from the background processes. Discrepancies between data and simulation of up to 50% are seen in the control region, depending on the phase space and the kinematic region. A reweighting factor is found by fitting a polynomial to the ratio of data to simulation in the control region and is applied to the background contribution in the signal region. The full difference between the background prediction with and without this reweighting is taken as a systematic uncertainty.

**Fig. 2:**
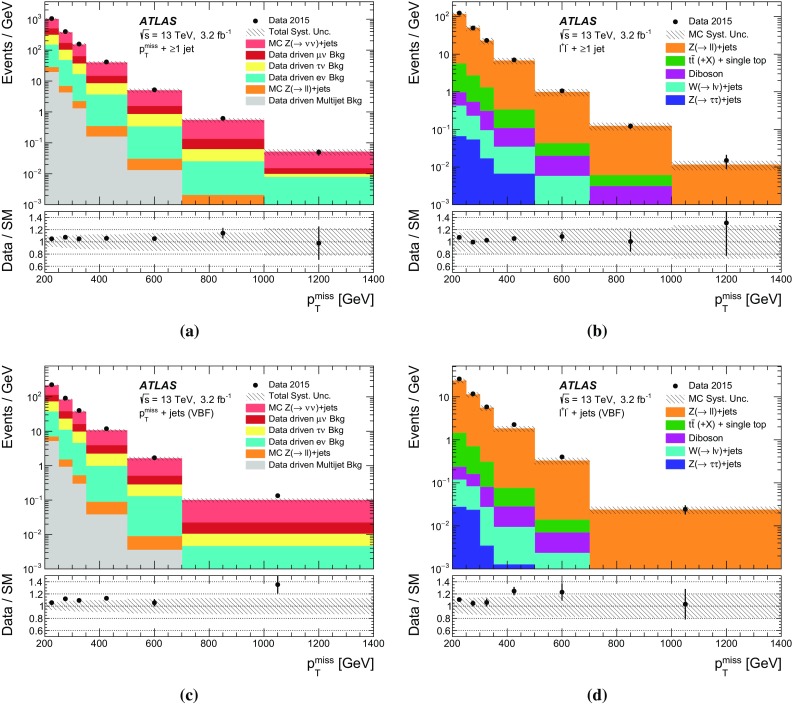
Comparisons between detector-level distributions for data and MC simulation of $Z \rightarrow \nu \bar{\nu }$ and  events plus predicted backgrounds in selected **a**, **c** $p_\mathrm {T}^\mathrm {miss}{} + \mathrm {jets}$ events and **b**, **d** $\ell ^{+}\ell ^{-} + \mathrm {jets}$ events as a function of the $p_\mathrm {T}^\mathrm {miss}$ variable in the **a**, **b** $\ge 1 \, \mathrm {jet}$ phase space and **c**, **d** VBF phase space. The lower panel shows the ratio of data to the Standard Model prediction. The error bars show the statistical uncertainty of the data. Uncertainties in the predictions are shown as hatched bands and include the statistical component as well as systematic contributions from theoretical predictions, lepton efficiencies and jet energy scales and resolutions to the MC predictions and uncertainties in the data-driven background estimates, explained in Sect. 8

**Fig. 3:**
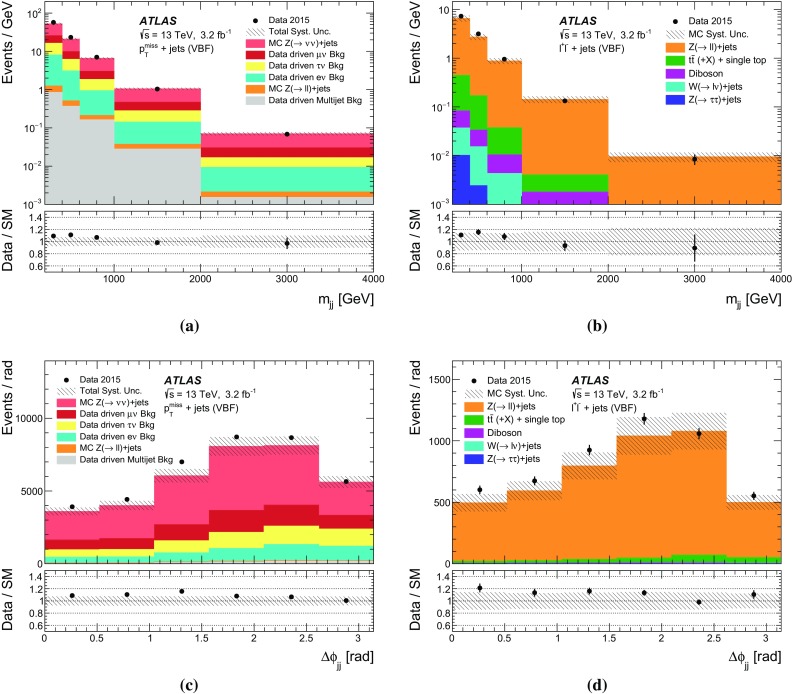
Comparisons between detector-level distributions for data and MC simulation of $Z \rightarrow \nu \bar{\nu }$ and  events plus predicted backgrounds in selected **a**, **c** $p_\mathrm {T}^\mathrm {miss}{} + \mathrm {jets}$ events and **b**, **d** $\ell ^{+}\ell ^{-} + \mathrm {jets}$ events as a function of **a**, **b** $m_{\mathrm {jj}}$ and **c**, **d** $\Delta \phi _{\mathrm {jj}}$ in the VBF phase space. The lower panel shows the ratio of data to the Standard Model prediction. The error bars show the statistical uncertainty of the data. Uncertainties in the predictions are shown as hatched bands and include the statistical component as well as systematic contributions from theoretical predictions, lepton efficiencies and jet energy scales and resolutions to the MC predictions and uncertainties in the data-driven background estimates, explained in Sect. 8

Figures 2 and 3 compare detector-level data to MC simulation of $Z \rightarrow \nu \bar{\nu }$ and  events, plus estimated backgrounds for selected $p_\mathrm {T}^\mathrm {miss}{} + \mathrm {jets}$ and selected $\ell ^{+}\ell ^{-} + \mathrm {jets}$ events in the signal region. Distributions of $p_\mathrm {T}^\mathrm {miss}$ in the $\ge 1 \, \mathrm {jet}$ and VBF phase spaces and for $m_{\mathrm {jj}}$ and $\Delta \phi _{\mathrm {jj}}$ in the VBF phase space are compared. For both the $p_\mathrm {T}^\mathrm {miss}{} + \mathrm {jets}$ and $\ell ^{+}\ell ^{-} + \mathrm {jets}$ event rates, the data are above the predictions from MC simulation and estimated backgrounds. However, they are consistent within the systematic uncertainties, which are discussed in Sect. 8 in more detail.

## Detector corrections

The data are corrected for the inefficiencies and resolutions of the detector and trigger and are presented in terms of particle-level variables as defined in Sect. 3. Due to the similarity in the $p_\mathrm {T}^\mathrm {miss}$ and jet selections between numerator and denominator, corrections for the $p_\mathrm {T}^\mathrm {miss}$ and jet-based variables arising from the jet energy resolutions and scales almost completely cancel in the ratio. Similarly, the correction factors related to the lepton veto efficiencies cancel in the ratio. The dominant remaining correction factor arises from the inefficiency of reconstructing the charged leptons in the denominator of the ratio. The correction factor is defined as the ratio of $R^{\mathrm {miss}}$ at particle level to $R^{\mathrm {miss}}$ at detector level using $Z \rightarrow \nu \bar{\nu }$ and $Z/\gamma ^{*} \rightarrow \ell ^{+}\ell ^{-}$ MC simulation, in bins of the measured variables. The correction factor decreases with $p_\mathrm {T}^\mathrm {miss}$ from 0.9 to 0.85 in the muon channel and increases with $p_\mathrm {T}^\mathrm {miss}$ from 0.7 to 0.8 in the electron channel. The number is larger for muons than for electrons because the reconstruction efficiency for muons is higher for the selection criteria used in this analysis.

Event migration between bins in the distributions, due to differences in the particle-level and detector-level variables, is small due to the relatively wide bins and therefore ignored. In the absence of a BSM signal, dependencies of the migrations on the underlying distributions are very similar for the numerator and denominator and therefore systematic uncertainties arising from this source cancel in the ratio. The possible impact of signals on the correction factors has been studied and found to be small. The presence of a large BSM component in the numerator due to WIMP production with an axial-vector mediator mass of 1 $\text {TeV}$ and a WIMP mass of 150 $\text {GeV}$ (which has very different event kinematics to the SM processes) changes the correction factor by less than 0.5%. The injected BSM model events have a $p_\mathrm {T}^\mathrm {miss}$ distribution that is much harder than the $Z \rightarrow \nu \bar{\nu }$ contribution to the numerator, leading to changes in $R^{\mathrm {miss}}$ of 4% at low $p_\mathrm {T}^\mathrm {miss}$ and 50% at high $p_\mathrm {T}^\mathrm {miss}$. Such a variation is much larger than the differences seen between data and SM simulation. Furthermore, injecting a Gaussian BSM contribution that adds events to a single bin (but remains consistent with the data) is also found to have a very small impact; the largest change in the correction factor is 2%, in the second bin of the $p_\mathrm {T}^\mathrm {miss}$ distribution, which is small compared to the systematic uncertainties. This test is an extreme example, where it is assumed that the full difference between the SM prediction and data in the $R^{\mathrm {miss}}$ ratio is due to BSM physics in the numerator. It is therefore concluded that the presence of any BSM model consistent with the data would lead to only small changes in the correction factors and that these models can be constrained by the detector-corrected results. Larger BSM contributions that could cause more significant changes in the correction factors have already been excluded with the detector-level data.

## Systematic and statistical uncertainties

Uncertainties in the measured detector-corrected ratios are discussed in this section and summarised in Table 2. The dominant experimental systematic uncertainties come from the reconstruction and isolation efficiency of muons and the reconstruction, isolation and trigger efficiency of electrons. These uncertainties affect the detector corrections, the $W$ background predictions from leptonic control regions and the backgrounds to $\ell ^{+}\ell ^{-} + \mathrm {jets}$ events. A smaller uncertainty in the $\tau$ reconstruction efficiency, affecting the $\tau$ veto, is also included. These are collectively labelled “Lepton efficiency” in the table. Uncertainties in the jet energy scale and resolution, labelled “Jets” in the table, affect the background predictions as well as the detector corrections. The latter arises due to small differences between the selected events for the numerator and denominator, such as the removal of jets close to leptons. The uncertainty from the difference in the choice of control region for the  background prediction, described in Sect. 6, is also included. For the multijet background estimation a 50% uncertainty in the number of predicted events, together with a smaller uncertainty found by varying the selection criteria for events used as input for the smearing method, is assumed. The difference between the reweighted and nominal MC simulation background prediction of $\ell ^{+}\ell ^{-} + \mathrm {jets}$ events is taken as an uncertainty. The reweighting factor is obtained from an $e^{\pm }\mu {\mp }$ control region, described in Sect. 6. Statistical uncertainties from the finite size of the MC simulation samples used to determine the detector corrections, in the $W$ control region data, and MC simulation samples used for extrapolations are also included.

Three categories of theoretical uncertainties are considered. Firstly, an uncertainty of 30% in the cross-section of processes involving top quarks in the numerator is assigned. This indirectly affects the extrapolation of $W$ events to the signal region by altering the number of top quark events in the control regions. The uncertainty value is motivated by top-quark-enhanced control regions constructed using the same criteria as the $W$ control regions but in addition requiring either one or two jets consistent with containing a *b*-hadron. Discrepancies between MC simulation and data of up to 30% are seen in these control regions, which justifies the large uncertainty. Secondly, theoretical uncertainties that affect the extrapolations between the control and signal regions for $W$ backgrounds are included. These are estimated by varying the factorisation, renormalisation, resummation scales (each scale varied by factors of 0.5 and 2) and the CKKW matching [32, 33] scale between 30 $\text {GeV}$ and 15 $\text {GeV}$ (the nominal being 20 $\text {GeV}$). These variations were found to affect the control and signal regions in the same way and the resulting uncertainties are therefore treated as fully correlated between the two. PDF uncertainties are derived for the nominal NNPDF3.0nnlo PDF set [35] as well as the MMHT2014 [73] and CT14 [74] PDF sets using their recommended PDF uncertainty prescription. A combined PDF uncertainty is then obtained from the envelope of the three PDF families and their respective uncertainties. An uncertainty from the strong coupling constant $\alpha _{\mathrm {S}} \left( m_{\mathrm {Z}}\right)$ is derived using up and down variations to 0.117 and 0.119, respectively (the nominal value being 0.118). Thirdly, the change in the $W$ background predictions when using Sherpa [28] v2.1.1 (which uses the CT10nlo [38] PDF set and has some technical differences in the parton shower compared to v2.2.0) or MG5_aMC@NLO v2.2.2 [40] instead of Sherpa v2.2.0 is considered. The second and third theoretical sources are included as “*W* theory” in Table 2. The correction factors do not change significantly when varying the SM MC event generator.

For each of the three data samples ($p_\mathrm {T}^\mathrm {miss}{} + \mathrm {jets}$, $e^+e^- + \mathrm {jets}$ and $\mu ^+\mu ^- + \mathrm {jets}$), the statistical uncertainty is taken as the Poisson error. For bins containing a small number of events, this uncertainty in the denominator leads to an asymmetric uncertainty in the ratio. Table 2 summarises the size of each systematic uncertainty and the statistical uncertainty from the data for the lowest and highest $p_\mathrm {T}^\mathrm {miss}$ bins in the $\ge 1 \, \mathrm {jet}$ phase space and the lowest and highest $m_{\mathrm {jj}}$ bins in the VBF phase space of the combined ratio. The uncertainties vary monotonically as a function of the respective observable.

**Table 2:** Summary of the uncertainties in the measured ratio $R^{\mathrm {miss}}$ for the lowest and highest $p_\mathrm {T}^\mathrm {miss}$ bins in the $\ge 1 \, \mathrm {jet}$ phase space and the lowest and highest $m_{\mathrm {jj}}$ bins in the VBF phase space. The statistical uncertainty is from the data. Statistical uncertainties in the MC simulation are included as systematic uncertainties. The uncertainties vary monotonically as a function of the respective observable

Systematic uncertainty source	Low $p_\mathrm {T}^\mathrm {miss}$ [%]	High $p_\mathrm {T}^\mathrm {miss}$ [%]	Low $m_{\mathrm {jj}}$ [%]	High $m_{\mathrm {jj}}$ [%]
Lepton efficiency	$+3.5$, $-3.5$	$+7.6$, $-7.1$	$+3.7$, $-3.6$	$+4.6$, $-4.4$
Jets	$+0.8$, $-0.7$	$+2.2$, $-2.8$	$+1.1$, $-1.0$	$+9.0$, $-0.5$
from control region	$+1.2$, $-1.2$	$+4.6$, $-4.6$	$+1.3$, $-1.3$	$+3.9$, $-3.9$
Multijet	$+1.8$, $-1.8$	$+0.9$, $-0.9$	$+1.4$, $-1.4$	$+2.5$, $-2.5$
Correction factor statistical	$+0.2$, $-0.2$	$+2.0$, $-1.9$	$+0.4$, $-0.4$	$+3.8$, $-3.6$
$W$ statistical	$+0.5$, $-0.5$	$+24$, $-24$	$+1.1$, $-1.1$	$+6.8$, $-6.8$
$W$ theory	$+2.4$, $-2.3$	$+6.0$, $-2.3$	$+3.1$, $-3.0$	$+4.9$, $-5.1$
Top cross-section	$+1.5$, $-1.8$	$+1.3$, $-0.1$	$+1.1$, $-1.2$	$+0.5$, $-0.4$
$Z\rightarrow \ell \ell$ backgrounds	$+0.9$, $-0.8$	$+1.1$, $-1.1$	$+1.0$, $-1.0$	$+0.1$, $-0.1$
Total systematic uncertainty	$+5.2$, $-5.2$	$+27$, $-26$	$+5.6$, $-5.5$	$+14$, $-11$
Statistical uncertainty	$+1.7$, $-1.7$	$+83$, $-44$	$+3.5$, $-3.4$	$+35$, $-25$
Total uncertainty	$+5.5$, $-5.4$	$+87$, $-51$	$+6.6$, $-6.5$	$+38$, $-27$

## Combination

After subtracting the estimated backgrounds from the selected $\ell ^{+}\ell ^{-} + \mathrm {jets}$ event sample in the data, and applying the bin-by-bin detector correction factor, the electron and muon denominators are combined using the best linear unbiased estimate (BLUE) combination method [75], which takes into account the relative precision of the two measurements. The technique correlates the statistical and systematic uncertainties between the two measurements and between all bins in all distributions. The combined result produces an average for $\ell ^{+}\ell ^{-} + \mathrm {jets}$ of one flavour in the denominator. The combination is iterated once, replacing the statistical uncertainty in the observed number of $Z \rightarrow \mu \mu$ and  events with that obtained from the expected number of events after the first combination. This removes the effect of undue weight being given to the channel in which the number of events has fluctuated down. In the combination, statistical correlations between bins are accounted for using a bootstrap method [76]. The  background uncertainty is assumed to be fully correlated or anti-correlated between bins, depending on whether the fit to estimate  background events increases or decreases the result from MC simulation in a given bin. The correlation between bins for the electron and muon efficiency uncertainties is found by considering the separate sources that contribute to the total uncertainties. All other sources of systematic uncertainty are assumed to be fully correlated across bins in the combination. The *p*-value for the compatibility of the two channels for all four distributions is 74%. The ratio is then formed by subtracting the estimated backgrounds from the selected $p_\mathrm {T}^\mathrm {miss}{} + \mathrm {jets}$ event sample in the data and dividing by the combined denominator. Again, each source of systematic uncertainty is assumed to be fully correlated between numerator and denominator. A cross-check using a maximum-likelihood fitting method gives consistent results.

## Results

**Fig. 4:**
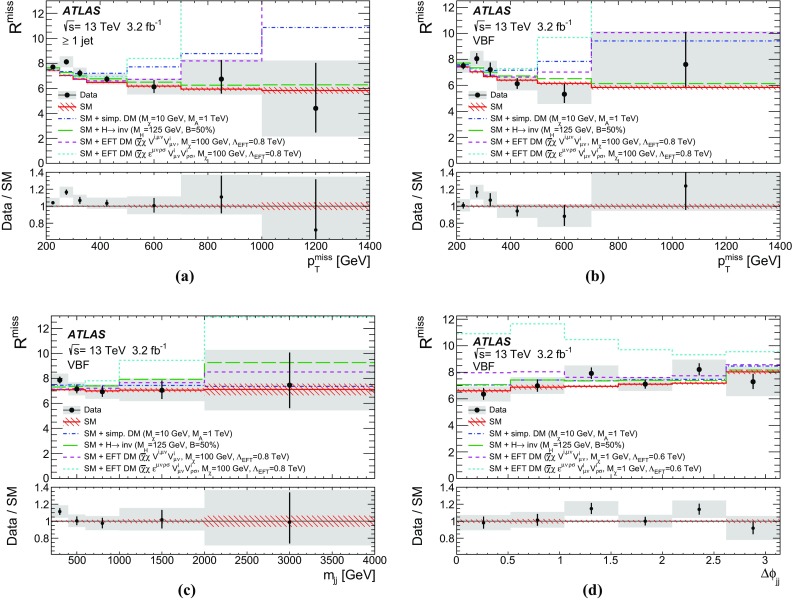
Measured $R^{\mathrm {miss}}$ as a function of **a** $p_\mathrm {T}^\mathrm {miss}$ in the $\ge 1 \, \mathrm {jet}$ region, **b** $p_\mathrm {T}^\mathrm {miss}$ in the VBF region, **c** $m_{\mathrm {jj}}$ in the VBF region and **d** $\Delta \phi _{\mathrm {jj}}$ in the VBF region. Statistical uncertainties are shown as error bars and the total statistical and systematic uncertainties are shown as solid grey bands. The results are compared to the SM prediction and to SM+BSM for four BSM models. One is a simplified model of WIMP production with an *s*-channel exchange of an axial-vector mediator with mass of 1 $\text {TeV}$ coupling to quarks and a WIMPs with a mass of 10 $\text {GeV}$, another represents the Higgs boson decaying to invisible particles with 50% branching fraction, and another two represent the predictions of two EFT operators allowing the production of WIMP dark matter through interactions with vector bosons (with differing charge-parity properties in the interaction). The $R^{\mathrm {miss}}$ values of the third and fourth models in the highest $p_\mathrm {T}^\mathrm {miss}$ bin in the $\ge 1 \, \mathrm {jet}$ region are 18.8 and 38.3, respectively, and in the highest $p_\mathrm {T}^\mathrm {miss}$ bin in the VBF region the fourth model has an $R^{\mathrm {miss}}$ value of 19.4. The red hatched error bars correspond to the uncertainty in the SM prediction. The bottom panel shows the ratio of data to the SM prediction

Figure 4 shows the four combined differential measurements of $R^{\mathrm {miss}}$ compared to the average of the Sherpa v2.2.0 SM particle-level predictions for the muon and electron channels. The measurement is consistent with the SM prediction within statistical uncertainties. The uncertainty in the SM prediction, found from the factorisation and renormalisation scale variations as well as the NNPDF3.0nnlo PDF uncertainty, explained in Sect. 8, is shown as a red hatched band in the figure. The SM predictions do not include NLO electroweak corrections beyond final-state photon radiation. These corrections were studied in Ref. [77] for the $Z$ boson production at a centre-of-mass energy of 8 $\text {TeV}$ and are very similar for the numerator and denominator with a residual effect of up to 1% on the ratio.

Also shown in the Fig. 4 is a comparison with SM+BSM for four BSM models. These four models comprise a simplified model for WIMP production with an *s*-channel exchange of an axial-vector mediator with a mass of 1 $\text {TeV}$ and a WIMP mass of 10 $\text {GeV}$, a Higgs boson decaying to invisible particles with 50% branching fraction, and two examples of effective field theory operators (each with different charge-parity properties) involving couplings of WIMP dark-matter candidates with vector bosons. These models are described in Sect. 5.

## Discussion

In Fig. 4a, b, both the measurements and the SM predictions show a ratio $R^{\mathrm {miss}}$ of approximately 7.5 at $p_\mathrm {T}^\mathrm {miss}{} = 200$ $\text {GeV}$, decreasing with $p_\mathrm {T}^\mathrm {miss}$ to approximately 6, which is very close to the SM ratio of branching fractions in the numerator and denominator of 5.9 [78].^3^ The ratio is larger at lower $p_\mathrm {T}^\mathrm {miss}$ values due to the fiducial requirements on the charged leptons in the denominator. At higher $p_\mathrm {T}^\mathrm {miss}$ values the leptons are more central and have larger $p_{\text {T}}$ , and are therefore more likely to pass the fiducial requirements. The removal of jets overlapping with charged leptons, described in Sect. 3, is only relevant to the denominator. In particular, a slight increase in the ratio towards large $\Delta \phi _{\mathrm {jj}}$ values is seen, indicating that jets with this topology are more likely to be removed in the denominator. The data and SM predictions are in agreement with an overall *p*-value including all distributions of 22% taking into account statistical and systematic correlations. In addition to the measured ratios, a covariance matrix for all four distributions, taking into account the statistical and systematic correlations between all bins in the data, is produced using a bootstrap procedure. When forming the covariance matrix the uncertainties are symmetrised by taking the maximum of the upward and downward uncertainties.

The detector-corrected ratio for all four distributions, together with the covariance matrix for the statistical and systematic uncertainties, as well as model uncertainties in the SM prediction for the numerator and denominator, and acceptance uncertainties in the WIMP model, are used to set limits on the mass of the axial-vector mediator ($m_A$) and WIMP candidate ($m_\chi$). Factors affecting the WIMP model signal acceptance include uncertainties in the modelling of initial- and final-state radiation in simulated samples, uncertainties in PDFs and the choice of $\alpha _{\mathrm {S}}\left( m_{\mathrm {Z}}\right)$, and the choice of renormalisation and factorisation scales.

Limits on dark-matter production models are set by first constructing the $\chi ^2$ function

$$\begin{aligned} \chi ^2 = {(\mathbf {y}_\mathrm {data} - \mathbf {y}_\mathrm {pred})}^{T} C^{-1} (\mathbf {y}_\mathrm {data} - \mathbf {y}_\mathrm {pred}), \end{aligned}$$

where $\mathbf {y}_\mathrm {data}$ and $\mathbf {y}_\mathrm {pred}$ are the vectors of the measured $R^{\mathrm {miss}}$ values and the predicted $R^{\mathrm {miss}}$ values for the hypothesis under test across the four distributions under study, *C* is the total covariance matrix defined as the sum of the statistical, experimental systematic and theoretical systematic covariances. The $\mathrm {CL}_\mathrm {s}$ technique [79, 80] evaluated using the asymptotic approximation [81] is used to derive upper limits.

**Fig. 5:**
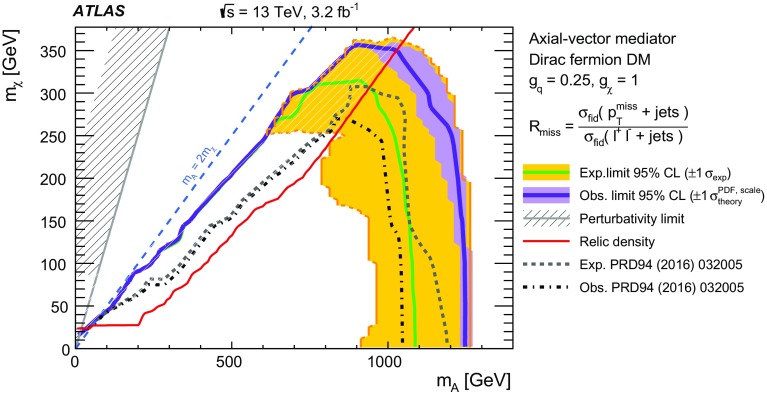
Exclusion contours (at 95% CL) in the WIMP–mediator mass plane for a simplified model with an axial-vector mediator and couplings $g_q = 0.25$ and $g_{\chi }=1$. The solid purple (green) curve shows the observed (expected) limit. The yellow filled region around the expected limit indicates the effect of $\pm 1\sigma$ experimental uncertainties in the expected limit. The red curve corresponds to the expected relic density. The grey hatched region shows the region of non-perturbativity defined by WIMP mass greater than $\sqrt{\pi /2}$ times the mediator mass. Also shown, for comparison, are limits set using detector-level event counts from Ref. [6]. The exclusion is based on the global fit to the $p_\mathrm {T}^\mathrm {miss}$ distributions in the $\ge 1 \, \mathrm {jet}$ and VBF phase spaces, and the $m_{\mathrm {jj}}$ and $\Delta \phi _{\mathrm {jj}}$ distributions in the VBF phase space

The overall rate and kinematic properties of events in the axial-vector mediator WIMP model under study are defined by four parameters: the WIMP candidate mass, the mediator mass and the strengths of the mediator interaction with quarks and WIMPs. The expected and observed 95% confidence level (CL) exclusion limits as a function of mediator and WIMP mass are shown in Fig. 5, for fixed mediator couplings of $g_q = 0.25$ and $g_\chi = 1$. Expected limits are shown with $\pm 1\sigma$ bands indicating the range of the expected limit in the absence of a signal. Observed limits are shown with a band including the effect of $\pm 1\sigma$ theoretical uncertainties in the WIMP model cross-section. Also highlighted is the region where perturbative unitarity is violated (where $m_\chi >\sqrt{\pi /2}\,m_A$) [82]. The points in the mass plane compatible with the relic density measured by Planck [83] and WMAP [84] are represented by a red continuous line, with WIMP masses below this line or mediator masses to the right of this line corresponding to dark-matter overproduction. The highest mediator mass observed (expected) to be excluded at 95% CL is 1.24 $\text {TeV}$ (1.09 $\text {TeV}$). For comparison, limits set using detector-level observables [6] are also shown. For high mediator masses, the expected limits in the present analysis are slightly weaker, due to the limited number of events in the denominator, whereas the observed limits are slightly stronger compared to the detector-level analysis. This difference between expected and observed limits is driven entirely by systematic uncertainty correlations between bins of the corrected distributions. Switching between using the default correlation model and a simple correlation model assuming 100% correlation between bins for each source of experimental systematic uncertainty changes the observed limit in mediator mass by approximately 10 $\text {GeV}$. The measurements presented in this paper have enhanced sensitivity to models with large WIMP masses and low mediator masses, with respect to the detector-level analysis presented in Ref. [6], due to the use of a larger fiducial volume and the use of differential information with associated correlations.

The detector-corrected data are also used to search for Higgs boson decays to invisible particles in the same manner. Limits are placed on the production rate of the Higgs boson multiplied by its branching fraction to invisible particles relative to the total Higgs boson production rate as predicted by the SM [85]. The expected 95% CL upper limit for a Higgs boson with a mass of 125 $\text {GeV}$ is found to be 0.59 with a range of [0.47, 1.13] from $\pm 1\sigma$ experimental uncertainties. The observed upper limit at 95% CL is 0.46. The most important distribution for setting limits in this model is $m_{\mathrm {jj}}$ , although some additional expected sensitivity is achieved from $\Delta \phi _{\mathrm {jj}}$ . The observed limits are stronger than expected due to systematic uncertainty correlations between bins in the corrected ratios. This is to be compared with an exclusion limit of 0.28 (0.31 expected) at 95% CL using a 20 fb$^{-1}$ 8 $\text {TeV}$ data set [12], with an event selection optimised for this particular process.

The detector-corrected data are further used to set limits on the production of Dirac-fermion dark matter in a generalised effective field theory (EFT) where dark matter interacts only with electroweak bosons. Limits are set as a function of the invariant mass of the dark-matter candidate and the EFT scale, $\Lambda$, which can be related to a UV-complete model by the relationship $1/\Lambda ^2 \sim g_\mathrm {SM}\, g_\chi /M^2$ where $g_\mathrm {SM}$ and $g_\chi$ would be couplings of the SM and dark-matter particles to some hypothetical heavy mediating particle with mass *M*. The scenario where production is dominated by two specific dimension-seven effective operators, $\bar{\chi }\chi V^{\mu \nu }V_{\mu \nu }$ and $\bar{\chi }\chi \varepsilon ^{\mu \nu \rho \sigma } V_{\mu \nu }V_{\rho \sigma }$, with differing *CP* properties in the interaction between two electroweak bosons ($V=W/Z$) and two dark-matter particles is considered. This EFT is described in Ref. [8] where an assessment of the EFT validity for these operators is also conducted. These operators are particularly interesting as sensitivity benchmarks since they are insensitive to constraints from *Z*-boson invisible-width measurements.

**Fig. 6:**
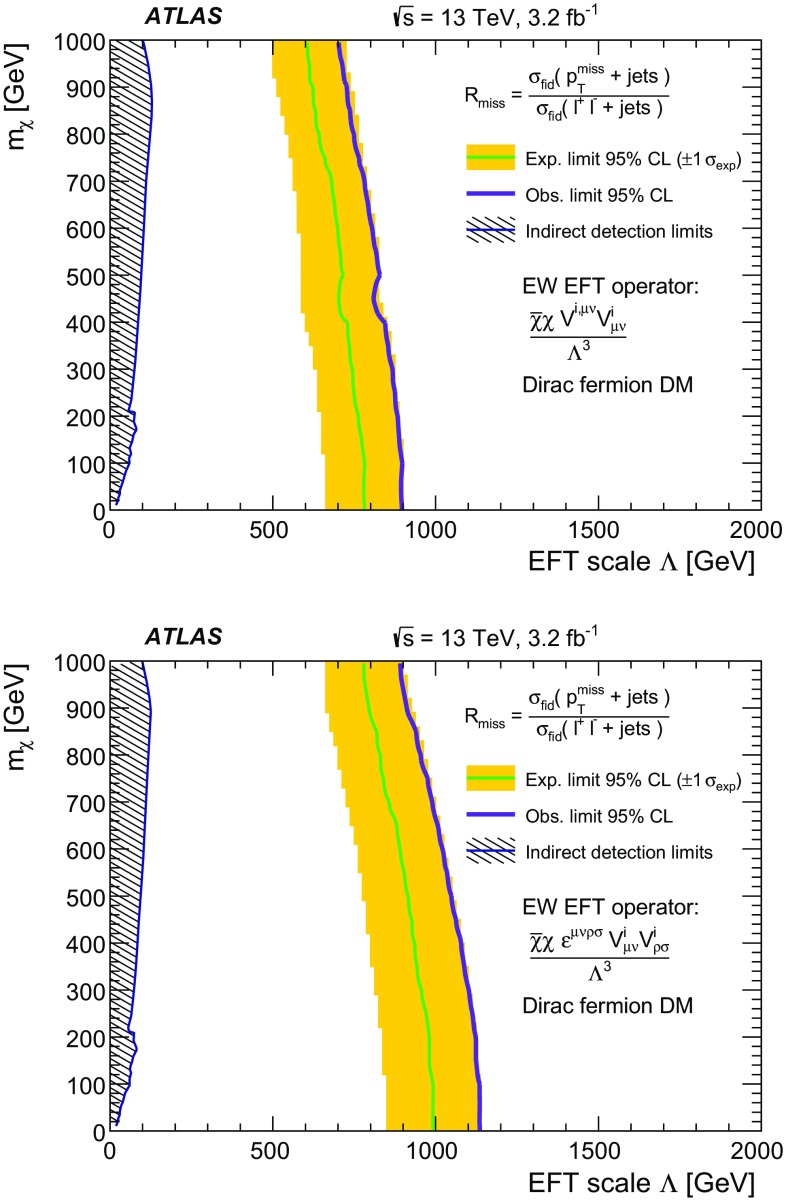
Exclusion contours (at 95% CL) for Dirac-fermion dark matter produced via a contact interaction with two electroweak bosons as described in an effective field theory with two dimension-seven operators (described in text) with different charge-parity properties. Limits are set as a function of dark-matter mass and the effective field theory scale, $\Lambda$. The solid purple (green) curve shows the median of the observed (expected) limit. Also shown are limits on these operators from indirect-detection experiments. The yellow filled region around the expected limit indicates the effect of $\pm 1\sigma$ experimental uncertainties in the expected limit. The exclusion is based on the global fit to the $p_\mathrm {T}^\mathrm {miss}$ distributions in the $\ge 1 \, \mathrm {jet}$ and VBF phase spaces, and the $m_{\mathrm {jj}}$ and $\Delta \phi _{\mathrm {jj}}$ distributions in the VBF phase space

Figure 6 shows the 95% CL expected and observed limits extracted from the fit to all four measured distributions, compared to indirect-detection limits. For the *CP*-conserving operator, expected (observed) limits on the EFT scale range from 0.78 (0.89) $\text {TeV}$ at low ($<200$ $\text {GeV}$) dark-matter mass to 0.61 (0.71) TeV at dark-matter masses of 1 $\text {TeV}$. Limits for the *CP*-violating operator are stronger than for the *CP*-conserving equivalent, ranging from 0.99 (1.14) $\text {TeV}$ at low dark-matter masses to 0.77 (0.89) $\text {TeV}$ at dark-matter masses of 1 $\text {TeV}$. Limits from indirect dark matter detection experiment results [8, 86, 87] interpreted in terms of these effective operators overlaid on Fig. 6 are sensitive up to EFT scales of 100–200 $\text {GeV}$.

The limits presented above assume a single operator would dominate the dark-matter production rate, but the detector-corrected data and covariance information can be used to explore more complex scenarios where multiple operators could contribute to the observed production rate with arbitrary relative rates and induce interference contributions between processes that would introduce non-trivial shapes and correlations between all three observables presented in this paper. The impact on the ratios in such an EFT model is demonstrated in Fig. 4 and is unlike the axial-vector mediator WIMP model and Higgs model presented above which predominantly modify only the $p_\mathrm {T}^\mathrm {miss}$ and $m_{\mathrm {jj}}$ distribution shapes, respectively.

The data have been corrected for detector effects and can be compared to any SM prediction or a combination of SM and BSM predictions at particle level, where the BSM model produces $p_\mathrm {T}^\mathrm {miss}{} + \mathrm {jets}$ final states. Models that also produce final states with at least one prompt lepton and $p_\mathrm {T}^\mathrm {miss}$ cannot be accurately compared to the data. This is because they will have been included in the $W$ background estimation, for which the extrapolation factors from control regions to the signal regions, determined using SM MC simulation, would be incorrect. Similarly, new-physics models with two leptons, entering the denominator, can only be reliably constrained by the data if the leptons have kinematics that are qualitatively similar to those in SM events, otherwise differences in the lepton efficiency correction factors may be observed. The data, together with the full covariance matrix for the uncertainties, are stored in HepData [88] and the analysis is included as a routine in the Rivet [89] software framework, in order to ease comparisons. Also stored in HepData are the SM numerator and denominator predicted by Sherpa, together with the covariance matrix for their uncertainties, such that these can be used when comparing to BSM models without having to simulate the SM contributions.

## Conclusions

Observables sensitive to the anomalous production of events containing one or more hadronic jets with high transverse momentum produced in association with a large $p_\mathrm {T}^\mathrm {miss}$ have been measured differentially with respect to a number of properties of the hadronic system. The results are presented as a measurement of the ratio of $p_\mathrm {T}^\mathrm {miss}{} + \mathrm {jets}$ to $\ell ^{+}\ell ^{-} + \mathrm {jets}$ events and are fully corrected for detector effects. This is the first detector-corrected measurement of observables specifically designed to be sensitive to dark-matter production.

The analysis uses 3.2 fb$^{-1}$ of proton–proton collision data recorded by the ATLAS experiment at the LHC at a centre-of-mass energy of 13 $\text {TeV}$. The results are presented in two phase-space regions defined by the hadronic system: a $\ge 1 \, \mathrm {jet}$ inclusive sample and a VBF topology. The particle-level differential ratio measurements are found to be consistent with the SM expectations.

Using this infrastructure, limits are placed in three BSM scenarios: a simplified model of pair production of weakly interacting dark-matter candidates, a model with an invisibly decaying Higgs boson, and an effective field theory with general interactions of electroweak bosons with a dark-matter candidate. Limits in simplified models are competitive with previous approaches and the use of shape information in the differential spectra measured in this paper provides improved sensitivity to models where the dark-matter candidate mass is close to half the mediator mass. For the specific effective field theory operators considered in the interpretation, the dark-matter interactions would evade direct-detection experiments. The results presented here represent the most stringent constraints to date on such interactions, with an order-of-magnitude improvement over previous limits from indirect-detection experiments.

The detector-corrected data are published along with the statistical and systematic uncertainty correlations so that they can easily be used in the future to place limits in a wide range of new-physics models that predict final states with jets and missing transverse momentum.

## Footnotes

1 ATLAS uses a right-handed coordinate system with its origin at the nominal interaction point in the centre of the detector and the *z*-axis along the beam pipe. The *x*-axis points to the centre of the LHC ring, and the *y*-axis points upward. Cylindrical coordinates $(r,\phi )$ are used in the transverse plane, $\phi$ being the azimuthal angle around the beam pipe. The pseudorapidity is defined in terms of the polar angle $\theta$ as $\eta =-\ln [\tan (\theta /2)]$. Rapidity is defined as $y=0.5 \ln [(E+p_z)/(E-p_z)]$ where *E* denotes the energy and $p_z$ is the momentum component along the beam direction.

2 Prompt refers to particles not coming from the decay of a hadron or from the decay of a $\tau$ lepton.

3 The denominator also includes the presence of the $\gamma ^*$ mediator, which is not present in the numerator and would influence the $R^{\mathrm {miss}}$ ratio in the SM; however, this contribution is small as the dilepton invariant mass is required to be close to the $Z$ mass.
